# When Do Band Gap Calculations Agree with Experiments in Monolayer-Protected Cu_14_ and Au_20_ Atomically Precise Nanoclusters? A (TD)-DFT Comparison of HOMO–LUMO, Fundamental, Optical, and Electrochemical Energy Gaps

**DOI:** 10.1021/acs.jpcc.6c01227

**Published:** 2026-04-13

**Authors:** Sarah Elhajj, Anik Sarkar, Yitong Wang, Rongchao Jin, Guoxiang Hu, Gangli Wang, Samer Gozem

**Affiliations:** † Department of Chemistry, 1373 Georgia State University, Atlanta, Georgia 30302, United States; ‡ Department of Chemistry, 6612 Carnegie Mellon University, Pittsburgh, Pennsylvania 15213, United States; § School of Materials Science and Engineering, 1372 Georgia Institute of Technology, Atlanta, Georgia 30332, United States

## Abstract

In view of the tremendous progress in atomically precise metal nanoclusters where electrochemical and optical energetics are routinely supported by computations to establish structure–function correlations, we explore the relationship between different protocols for measuring and computing band gaps of two distinct organic ligand-protected nanoclusters: [Cu_14_H_10_(MBN)_3_(PPh_3_)_8_]^+^ and Au_20_(TBBT)_16_. Through UV/visible spectroscopy and differential pulse voltammetry, we measure optical and electrochemical band gaps in those systems. We then compare these experimentally determined gaps to HOMO–LUMO gaps, fundamental gaps, vertical excitation energies, and *E* ^o^_ox_ *– E* ^o^_red_ potentials computed using different density functional theory (DFT) or time-dependent DFT (TD-DFT) methods. Specifically, in both copper and gold nanoclusters, we test the effect of truncating inert ligands from the model and compare density functionals with varying degrees of Hartree–Fock (HF) exchange from 0 to 50%, range-separated hybrids with a varying long-range tuning parameter, different correlation functionals, basis sets, and (equilibrium and nonequilibrium) continuum solvation models. Despite having different frontier orbital characters (the copper nanocluster has a metal-to-ligand charge transfer character while the gold nanocluster has metal-centered frontier orbitals), both nanoclusters display a similar sensitivity of the HOMO–LUMO gap to the HF exchange that is partially mitigated when computing the fundamental, optical, and electrochemical gaps. Other factors, such as the nature of the correlation functional, basis set, and geometry relaxation, have a considerably smaller effect on computed band gaps in these systems. Overall, this work provides guidelines for factors of varied importance for correlating computed and experimental band gap values.

## Graphical abstract

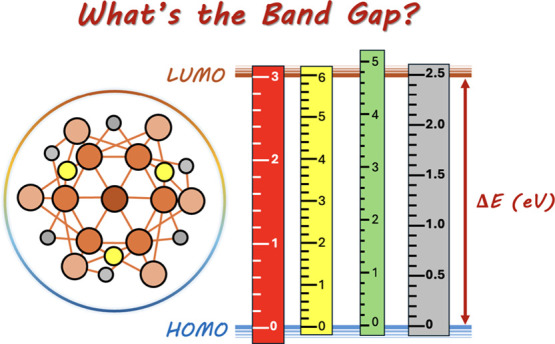

## Introduction

A material’s band gap is a fundamental quantity that is closely connected to its optical, redox, and other electronic transport properties. The ability to reliably and rapidly predict band gaps in materials of varying length scales would accelerate the development of novel materials with a wide range of applications in areas ranging from electro- and photocatalysis and photovoltaics to bioimaging and sensing.^1–3^

Modern quantum chemical electronic structure methods can predict the relative energetics of ground, excited, and ionized states of small molecules with subkilocalorie per mole accuracy.^4,5^ However, the exponential scaling of computational cost with the size of nonperiodic systems often necessitates the use of approximate methods. Strongly correlated systems, such as metal oxides, magnetic materials, and other quantum materials, still pose a challenge to routine computation since they require methods that often scale unfavorably with system size.^6^ However, systems with closed-shell electronic character or that do not have strong multiconfigurational character are more tractable because they can be described reasonably well using single-reference methods.

Atomically precise nanoclusters (APNCs) represent intermediate cases that maintain some bulk-like material properties while still having molecule-like discretized orbital energies. Such materials possess a metal core of a specific number of metal atoms and an exterior ligand shell, and are represented by exact molecular formulas, with their atomic structures determined by single crystal X-ray diffraction.^7–17^ A popular choice of electronic structure theory for APNCs is Density Functional Theory (DFT). However, even APNCs with closed-shell electronic character require the use of balanced approximations. Large errors can render the computations too unreliable even to be qualitatively useful.

Band gaps in materials are defined as energy differences between the edge of the conduction band and the edge of the valence band. In a perspective article titled “*Mind the Gap*!”,^18^ Bredas clarifies the distinction between different uses of the term “band gap” in the context of experimentally measured or computationally evaluated energy gaps. In APNCs and organic materials, the definition of band gap is extended to describe the energy gap between the highest occupied molecular orbital (HOMO) and the lowest unoccupied molecular orbital (LUMO). When approximate DFT or wave function methods are used, the HOMO–LUMO gap is generally understood to be a convenient approximation to one of several energy gaps that can be probed experimentally.

Here, we explore different approximations employed computationally in relation to experimentally measured electrochemical and optical gaps. We start with the simplest approximation, the HOMO–LUMO gap (Figure 1a), and then compute the fundamental gap (Figure 1b), optical gap (Figure 1c), and electrochemical gap (Figure 1d). We explore the role of Hartree–Fock (HF) exchange and long-range separated hybrids in DFT functionals, as well as different correlation functionals, basis sets, and solvation effects, on those energy gaps.

**Figure 1:**
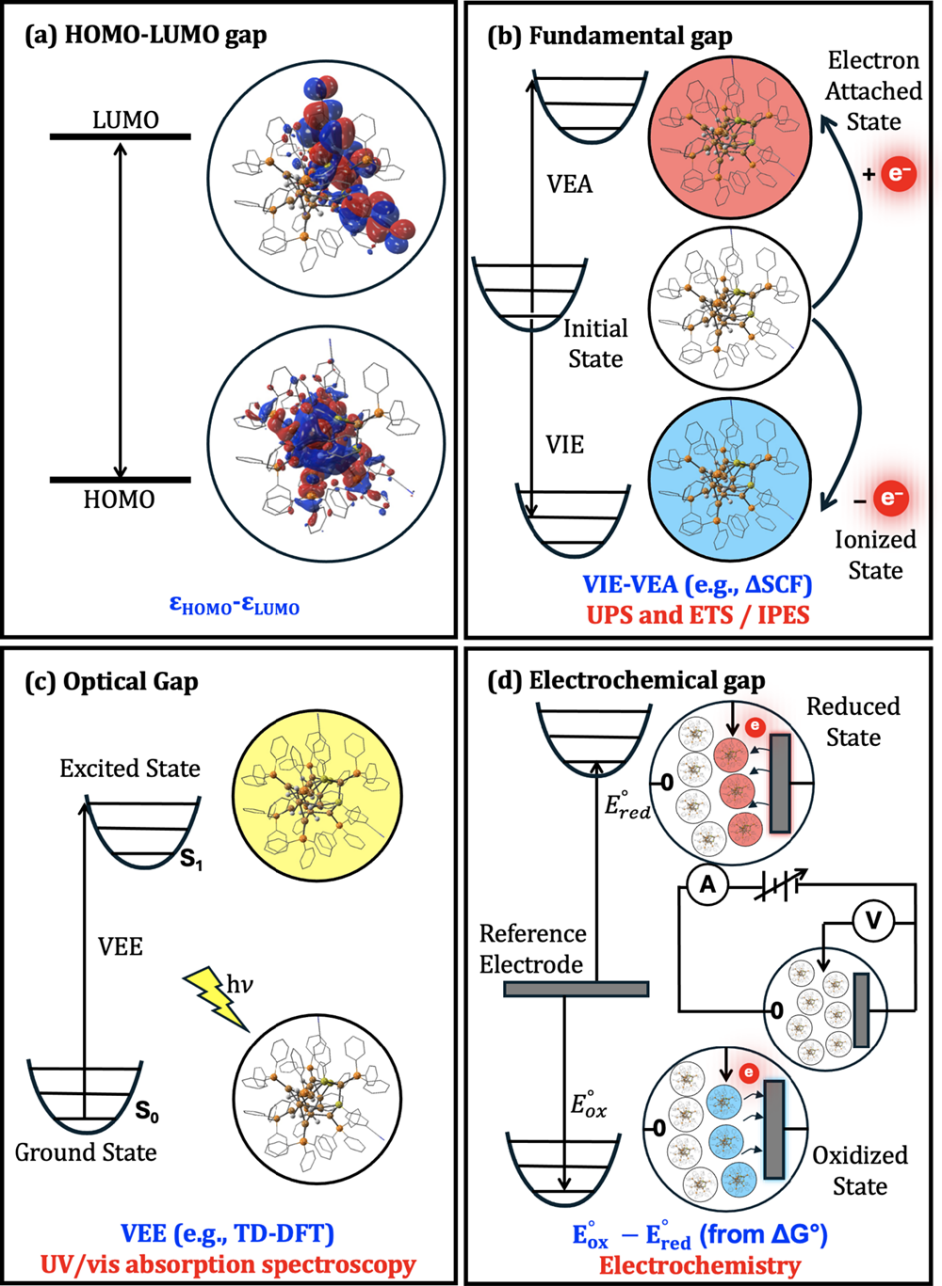
Schematic illustration of four different energy gaps; (a) HOMO–LUMO gap, (b) fundamental gap, (c) optical gap, and (d) electrochemical gap. At the bottom of each panel, a computational approach to determine the energy gap is indicated in blue, while a corresponding experimental approach typically used to measure the energy gap is in red. The HOMO–LUMO gap’s relation to the other energy gaps is discussed in the Theoretical Background section. The encircled structures shown in each panel without a background color represent the ground state, while the structures with yellow, red, and blue background represent an electronic excited state, a reduced or electron attached state, and an oxidized or ionized state, respectively. Acronyms: VIE, vertical ionization energy; VEA, vertical electron attachment; ΔSCF, Delta self-consistent field; UPS, Ultraviolet photoelectron spectroscopy; ETS, Electron transmission spectroscopy; IPES, Inverse photoemission spectroscopy; VEE, vertical excitation energy; TD-DFT, time-dependent DFT; UV/vis, Ultraviolet/visible; *E*_ox_°, standard oxidation potential; *E*_red_°, standard reduction potential; Δ*G* °, change in Gibbs free energy.

We focus on two representative nanoclusters: [Cu_14_H_10_(MBN)_3_(PPh_3_)_8_]^+^ and Au_20_(TBBT)_16_ with (MBN = 4-mercaptobenzonitrile; PPh_3_ = triphenylphosphine; TBBT = 4-tertbutylbenzene thiol or SPh-*t*-Bu). In the remainder of this work, these nanoclusters will be referred to as Cu_14_ and Au_20_, respectively. Cu_14_, which was selected due to its small size, belongs to a relatively new class of copper hydride APNCs. This specific cluster was recently synthesized and characterized both experimentally and computationally.^19^ Its first excited state was found to have metal-to-ligand charge transfer (MLCT) character. The second system is among the smaller members belonging to a widely studied class of thiol-functionalized gold APNCs.^7,9,11,20–24^ This system instead has a first excited state that is localized on the metal core. Those two systems are different with respect to the nature of their metallic cores, ligands, and electronic character of the first excited state. Therefore, the results of the two distinct nanoclusters indicate that key conclusions in this work are useful for related types of small- to medium-sized nanoclusters.

## Theoretical Background

### The Meaning of Orbitals

While the terms HOMO and LUMO are embedded in the chemistry literature, it may be useful to briefly review some general concepts related to molecular orbitals (MOs). First, while atomic orbitals for a 1-electron hydrogen-like atom are exact solutions (eigenfunctions) to the Schrödinger equation and have clearly defined energies (eigenvalues) and shapes, MOs are not unique eigenfunctions of a multielectron Schrödinger equation. Instead, they are eigenfunctions of one-electron operators constructed to describe the spatial distribution of an electron in a molecule’s field, and therefore they only gain meaning or relevance to experiments from the way they are solved.^25^ Different experimental techniques probe different orbitals. For example, canonical molecular orbitals (within Koopmans’ theorem, *vide infra*) or Dyson orbitals are associated with photoelectron spectroscopies,^25–28^ while natural transition orbitals (NTOs) are associated with electronic transitions (i.e, excitations, de-excitations).^29,30^ Two-photon absorption NTOs are distinct from one-photon NTOs.^31^ Natural bond orbitals, natural localized molecular orbitals, and natural hybrid orbitals can be used to extract useful chemical information like bond order and Lewis structures.^32–35^ As written by Krylov in *“From orbitals to observables and back”*: “Different experiments probe different types of orbitals—hence, what you see depends on the tool you use to interrogate your system. This lack of a single definition of the true orbitals is not a flaw of the theory; rather, it is fully in accord with the quantum mechanical world in which the probe and the system that is probed are interconnected.”^25^

Over-reliance on one set of MOs to explain different chemical and electronic properties of a system may be misleading. A simple but classical example is H_2_O; water’s 10 electrons occupy 5 molecular orbitals that appear as five separate bands in its photoelectron spectrum, consistent with water’s canonical molecular orbitals obtained from a HF calculation.^36,37^ This may appear inconsistent with the two equivalent “rabbit ear” lone pairs of water derived from VSEPR theory. However, the latter better illustrates water’s ability to form two equivalent H-bonds in a tetrahedral geometry. Orbitals consistent with VSEPR theory can be obtained by using a different linear combination of MOs instead of the canonical ones and are more useful for understanding water’s H-bonding behavior than the canonical molecular orbitals.^36–38^

### Koopmans’ Theorem

Within the HF approximation, *canonical* MOs diagonalize the Fock matrix and have eigenvalues (orbital energies) equal to the negative of the ionization potential.^39^

ϵ=−IE 1

When the terms HOMO and LUMO are used in HF, they refer to the highest occupied and lowest unoccupied canonical molecular orbitals, respectively; HOCMO and LUCMO seem like more descriptive acronyms, (where, “C” is to indicate “canonical”), but they are never used. Due to the connection to ionization energies, HOMO and LUMO energies can be directly connected to a first ionization energy (IE) measured by ultraviolet photoelectron spectroscopy (UPS) and a first electron affinity (EA) measured by electron transmission spectroscopy (ETS), respectively.^40^ In the solid state, inverse photoemission spectroscopy (IPES) is used to measure electron affinity instead of ETS.^41^ While UPS and ETS/IPES are limited to gas/solid surface states, developments such as microjets have enabled their use for probing systems in the liquid or solution phase.^42,43^

The difference between IE and EA is termed the fundamental gap (Figure 1b).^44–46^ The equality in eq 1, termed Koopmans’ theorem,^39^ inherits the missing electron correlation from the methodology (i.e., electron–electron interactions treated with the mean-field approach in HF). Therefore, HOMO–LUMO gaps obtained from HF are only approximations to the fundamental gap. There have been numerous discussions and derivations extending Koopmans’ theorem to correlated wave functions^47^ and Kohn–Sham MOs obtained from exact density functional theories.^48–51^ For instance, Koopmans’ theorem has been shown to be satisfied for density functionals that better address delocalization (self-interaction) error.^52,53^ “Koopmans’ compliance” is increasingly being used as a check for the accuracy of DFT methods, and in turn to tune DFT parameters such as HF exchange and long-range correction parameters to enforce Koopmans’ theorem.^44,45,54–63^ Koopmans’ compliant functionals can yield canonical Kohn–Sham orbitals that better resemble Dyson orbitals,^26,64,65^ and may potentially have benefits that translate to the calculation of properties other than ionization energies. In the absence of such calibrations, one should not expect that either HF or Kohn–Sham DFT HOMO and LUMOs will give quantitative agreements with experimentally determined fundamental gaps.

### Fundamental Gap Using the ΔSCF approach

More accurate fundamental energy gaps can be obtained from approximate DFT methods by separately computing the energy of the system in its each of its initial ground state, ionized state, and electron attached state. The differences between those energies (ΔSCF) provide ionization and electron attachment energies that can then be used to compute the fundamental gap.^66^

### The Vertical Approximation

A complication arises when comparing a single-valued energy gap to experiments; experimental energy gap measurements are broadened by vibrational, rotational, and other factors. Accurate simulations would require accounting for nuclear motion, which can be achieved by molecular dynamics to classically sample vibrational energies, or by the calculation of Franck–Condon factors.^27,67–69^ On the other hand, the use of vertical ionization energy (VIE) and vertical electron affinity (VEA) is a very common approximation consistent with the Franck–Condon Principle (i.e., since excitation is fast relative to nuclear rearrangement). Using this vertical approximation, the energy of the initial and ionized states are computed at a single geometry representing the initial state.

### Optical Gap Using TD-DFT

A system’s “band gap” may be probed spectroscopically in the UV, visible, or near-IR range (depending on the magnitude of the gap). Such an energy gap is more suitably termed the optical gap (see Figure 1c).^18,44^ Often, a Tauc plot is used by extrapolating the linear region of the lowest energy absorbance band vs photon energy plot to find the photon energy axis intercept. While the fundamental gap refers to removal/addition of an electron to the HOMO/LUMO, the optical gap involves promoting an electron from the HOMO to the LUMO. The nature of those two energy gaps is different; electron affinity involves a Coulombic penalty associated with one extra electron–electron repulsive interaction. Therefore, generally, the optical gap is expected to be lower than the fundamental gap.^18^

The optical gap is only relevant to probing a system’s HOMO–LUMO gap if the first excited state is optically active. If the HOMO → LUMO optical transition is symmetry-forbidden or has a very low transition dipole moment, then it does not appear in a UV/visible spectrum. In such a case, the Tauc plot reveals a photon energy associated with a higher energy transition such as one dominated by (HOMO → LUMO + m) or (HOMO-n → LUMO) character.

Optical gaps can be obtained computationally using an excited-state electronic structure method such as time-dependent DFT (TD-DFT).^70–73^ Again, the effect of nuclear motion can be incorporated through molecular dynamics or the calculation of Franck–Condon factors for a better comparison between a simulated spectrum and the experimental UV/vis one, but the vertical approximation is also widely employed due to the instantaneous nature of excitation. The vertical excitation energy (VEE) is therefore simply the difference in energy between the S_0_ and S_1_ states computed at equilibrium geometry of the ground S_0_ state (Figure 1c).

### Electrochemical Gap from Δ*G* Calculations

A system’s band gap may be probed electrochemically through the measurement of reduction and oxidation potentials (electrochemical gap, see Figure 1d).^74,75^ Experimentally, the redox potentials can be measured by one of several methods such as differential pulse voltammetry (DPV).^15,16,76^ The electrochemical gap is distinct from the fundamental and optical band gaps because it involves equilibrium rather than instantaneous (nonequilibrium) processes. Electrochemical potentials are related to free energies:

$$E^{{}^{\circ}}=-\frac{\Delta G^{{}^{\circ}}}{nF}$$ 2

$$E^{{}^{\circ}}=E_{\mathrm{ox}}^{{}^{\circ}}-E_{\mathrm{red}}^{{}^{\circ}}$$ 3

The computation of electrochemical band gaps requires accounting for the geometric relaxation of the system upon reduction/oxidation and the inclusion of additional terms such as zero-point vibrational energies, pressure–volume work, and entropy.^77,78^ Computationally, entropy can be estimated from translational, rotational, vibrational, and electronic contributions to the system’s partition function. The vibrational contributions require the calculation of vibrational frequencies (e.g., using DFT).

### The Effect of the Solvent

The above discussion has avoided the topic of solvation so far. Experimentally, it is not easy to isolate the effect of solvation for complex systems such as APNCs, since that typically would require a comparison to a reference such as gas-phase measurements. However, solvation effects can be much more easily predicted computationally.

Figure 2 represents different ways to model a solvent’s response to an electronic change in the solute. Here, ionization of the solute (from a neutral state to a positively charged state) is shown as a representative example. When modeling UV/vis and photoelectron spectra (e.g., using VEE or VIE/VEA calculations), the vertical approximation means that neither the solvent nor the solute nuclear coordinates have time to relax on the time scale of the electronic transition.^79^ In this case, it may be suitable to model the solvent as a static environment that is not affected by the solute’s excitation (see Figure 2a). This level of approximation is used in hybrid quantum mechanical/molecular mechanical (QM/MM) methods where the solvent is treated as a set of point charges. In reality, excitation or removal/addition of an electron on the solute polarizes the solvent’s electrons, which can be captured through nonequilibrium solvent models that account for solvent polarization without reorienting its permanent dipoles (see Figure 2b). Computationally, this can be achieved using a polarizable explicit solvent model^80^ or, more conveniently, by using a nonequilibrium polarizable continuum model (ne-PCM).^79,81^

**Figure 2:**
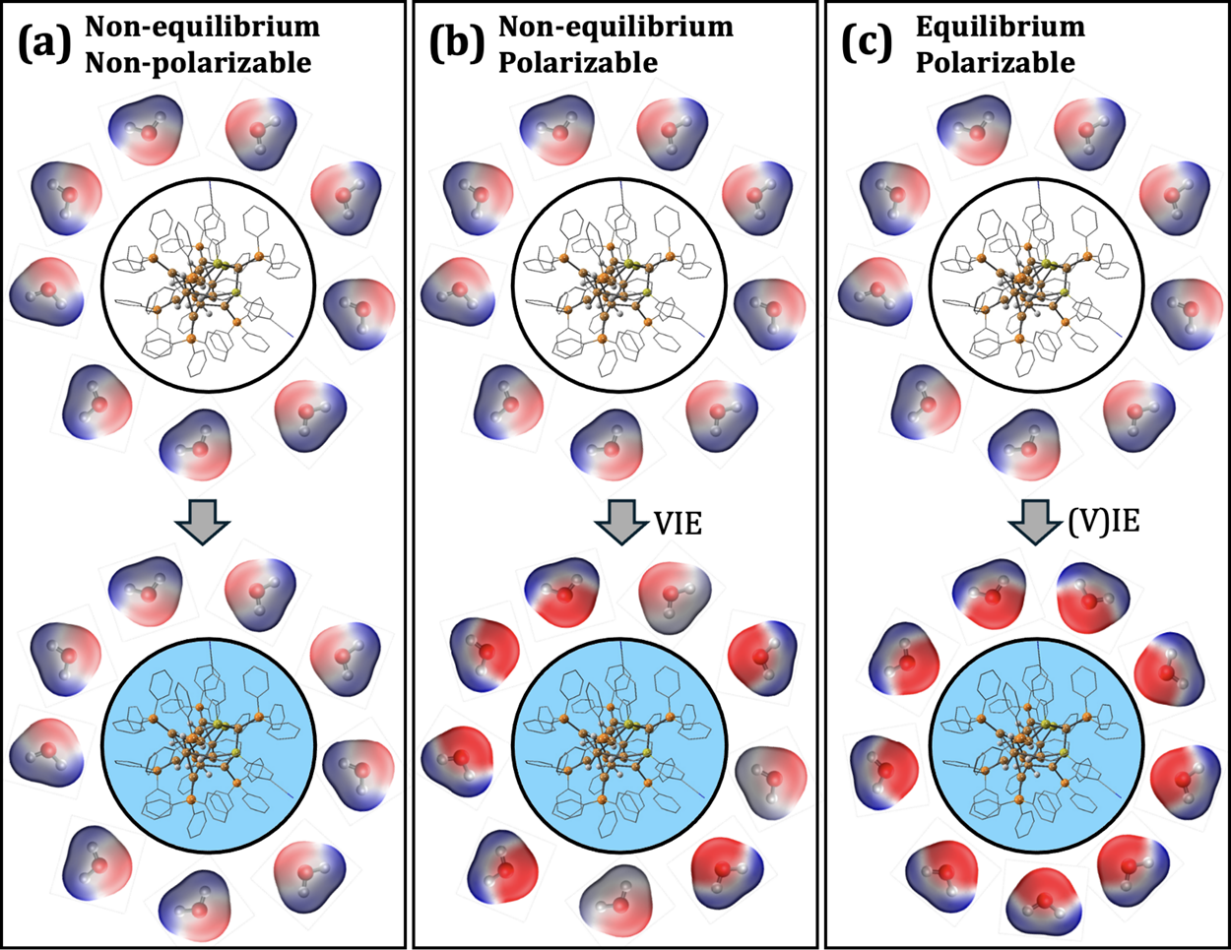
Schematic illustration of (a) non- equilibrium, nonpolarizable (left panel), (b) nonequilibrium, polarizable (center panel) and (c) equilibrium, polarizable (right panel) solvation models in response to ionization of a solute. A fourth possibility, equilibrium nonpolarizable solvation, is not shown here. While this scheme uses an atomistic representation of water, a polarizable continuum model (PCM) can be used to compute the nonequilibrium (ne-PCM) and equilibrium (e-PCM) solvation effects, analogous to what is shown in panels (b) and (c), respectively.

In the case of slow (equilibrium) processes such as redox chemistry, the computational model should account for relaxation of the solvent in terms of nuclear and electronic degrees of freedom (see Figure 2c). For an explicit solvent model (e.g., QM/MM or a model that includes the water molecules in the QM calculation), this would require rearrangement of the solvent molecules in response to the solute’s excited or ionized electronic state. Alternatively, this solvation effect can be captured using equilibrium PCM (e-PCM, see Figure 2c).^79,82^ In this work, we do not use QM/MM explicit solvent models and instead use PCM to capture the effect of solvation. Specifically, we employ both ne-PCM (which is analogous to Figure 2b) and e-PCM (which is analogous to Figure 2c).

### The (V)­IE-(V)­EA Approximation

The electrochemical gap can be approximated using a fictitious e-PCM (V)­IE-(V)­EA approach, where the vertical IE and EA are computed using e-PCM rather than ne-PCM solvation. We use the parentheses for (V)­IE-(V)­EA to indicate that those are not true vertical ionization/electron affinity energies but instead account for solvent relaxation through the use of e-PCM as opposed to VIE-VEA energies that are computed with nonequilibrium solvation. The (V)­IE-(V)­EA approach neglects nuclear relaxation of the solute, as well as free energy terms like entropy; only the solvent relaxation, introduced by using e-PCM instead of ne-PCM, is included to provide a useful approximation to redox free energies. When using this approximation, the other terms like entropy and solute relaxation are assumed to be similar for the oxidized, reduced, and neutral state.

### Overview of This Section and Goal of This Work

The Theoretical Background section above provides a brief summary of different ways of computing band gaps and some of the widely used approximations. A substantial number of benchmark studies have investigated, for instance, the relationship between HOMO–LUMO gap and fundamental gap, optical gap, or electrochemical gap. However, it is rare to see all these energy gaps computed at the same time.^83–86^ Furthermore, while the HOMO–LUMO gap is typically connected to the fundamental gap through Koopmans’ theorem, we find that this energy gap displays a much stronger dependence on solvation effects than, for instance, the optical gap. How, in this case, should range-separated or exchange tuning be done?

In this work, we explore such questions in APNCs, which are interesting test cases because their electronic structure bridges molecular and bulk-like properties. The calculations are compared to energy gaps measured experimentally. Since fundamental gaps are difficult to measure for solvated molecules, the electrochemical band gaps are measured instead, in addition to the optical gap (if the HOMO–LUMO transition is bright).

## Experimental Methods and Results for Cu_14_ and Au_20_

The experimental details for the synthesis, optical characterization, and electrochemical characterization for the two nanoclusters investigated in this work are presented in Section S1 of the Supporting Information (SI) document.

### Cu_14_ Electrochemistry

The electrochemical and optical gap of Cu_14_ and Au_20_ nanoclusters were experimentally determined using DPV and UV/visible spectroscopy, respectively. The Cu_14_ nanocluster exhibited an oxidation peak at 0.35_6_ V and a reduction peak at −1.71_7_ V in the DPV, giving an electrochemical bandgap of 2.07_3_ eV [0.35_6_ – (−1.71_7_)], as shown in Figure 3a (see also Table S1). The reduction feature at −1.71_7_ V vs SHE was weakly resolved, especially in the oxidation scan which always starts at more negative potentials, −1.9 V herein. This is due to the stability of the reduced Cu_14_ and limited electrochemical window of methylene chloride, which becomes unstable beyond approximately – 1.8 V vs SHE (cyclic voltammograms (CV) in Figure S1). One additional oxidation peak is resolved at +0.69_4_, corresponding to 2*e* oxidation (forming Cu_14_^3+^). This should not be simply assumed to be a larger charging energy of 0.33_8_ eV, albeit an increase in charging energy with a decrease in metal core size is qualitatively consistent with concentric sphere model.^87,88^ The reason is that a shift in molecular orbital energies (or split of originally degenerate orbitals) can not be ignored. The charging energy and its effect on redox potentials is discussed in more detail in the Results and Discussion section.

**Figure 3:**
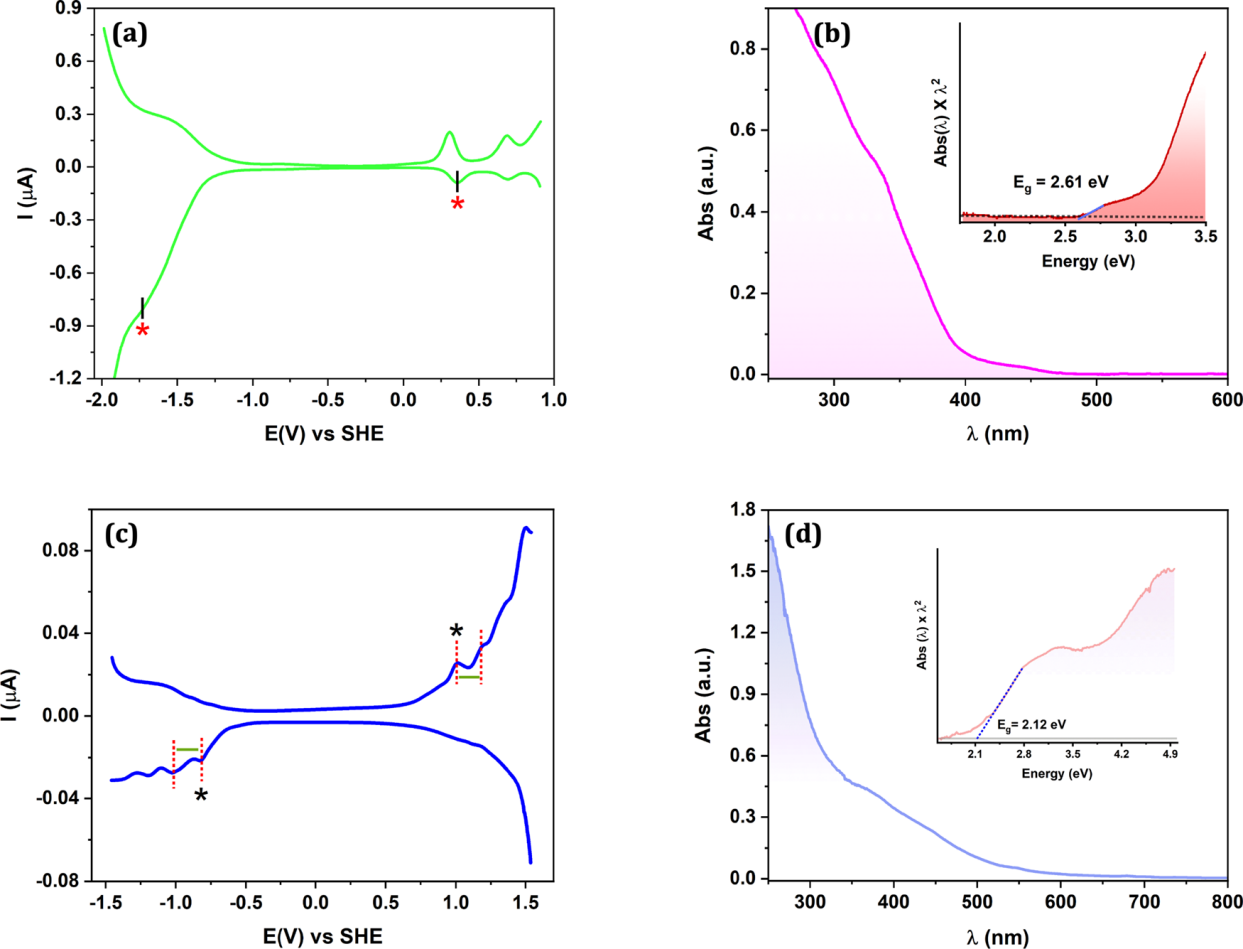
(a) Differential Pulse Voltammetry (DPV) and UV–visible absorption analysis (a,b) for [Cu_14_H_10_(MBN)_3_(PPh_3_)_8_]­BF_4_] and (c,d) for Au_20_(TBBT)_16_. Data are recorded in Dichloromethane. The insets in UV–vis spectra plot the absorbance in Energy scale. Blue dashed lines illustrate the extrapolation to determine the optical HOMO–LUMO gap of 2.61 and 2.12 eV in (b) and (d), respectively. The potential is referenced against Standard Hydrogen Electrode Potential (SHE).

### Cu_14_ UV/Vis Absorption

The UV–Vis absorption spectrum of Cu_14_ (Figure 3b) displayed shoulder bands at 295 and 332 nm with a weak band around 443 nm, yielding an apparent optical bandgap of 2.61 eV or 475 nm (blue intercept at the inset of Figure 3b). However, as noted later, the first excited state of Cu_14_ is dark, and therefore the 2.61 eV is not representative of the HOMO–LUMO gap and rather is associated with excitation to a higher energy state.

### Au_20_ Electrochemistry

For Au_20_, the DPV in Figure 3c reveals an oxidation peak at 1.00_6_ V and a reduction peak at −0.82_0_ V (Table S1), giving an electrochemical energy gap of 1.82_6_ eV [(1.00_6_ – (−0.82_0_))]. Weak or absent differential current peaks in the negative potential range during the oxidation scan and in the positive range during reduction scan is due to instability of the NCs (CVs in Figure S1). The wavy pattern following the first major oxidation and reduction is attributed to charging energy effects, estimated at 0.18_6_ V from the peak spacing illustrated by the green bar.

### Au_20_ UV/Vis Absorption

The UV–Vis absorption spectrum of Au_20_ (Figure 3d) displays shoulder bands at 371, 445, and 546 nm, from which the optical gap was determined as 584 nm or 2.12 eV (blue intercept at the inset of Figure 3d).

## Computational Methods

### Model Construction

Input geometries for the copper and gold nanoclusters were obtained from the crystal structures. While several calculations were carried out for the full [Cu_14_H_10_(MBN)_3_(PPh_3_)_8_]^+^ model, most calculations are carried out for a truncated model where the inert triphenylphosphine PPh_3_ ligands were replaced with PH_3_ to reduce the cost of calculations, yielding [Cu_14_H_10_(MBN)_3_(PH_3_)_8_]^+^. Similarly, for Au_20_(TBBT)_16_, the bulky 4-*tert*-butylbenzenethiol (TBBT) ligands were replaced with methylthiolate (SCH_3_). The practice of truncating nonconjugated segments of ligands to reduce computational cost has been adopted earlier in numerous studies.^89–92^ The effect of such a truncation is discussed in the Results and Discussion section.

Geometry optimizations were carried out at the Perdew–Burke–Ernzerhof (PBE)^93^ level of theory with the def2-SVP basis set unless specified otherwise. In the case of Au atoms, the def2-SVP effective core potential (ECP) is used.^94^ All optimized structures were verified to be stationary points by frequency calculations; that is, the studied molecules have no imaginary frequencies. Excited-state energies were computed using TD-DFT with the same model chemistry as the ground state calculations.^72^ To account for solvation effects, the implicit PCM solvation model was employed using dichloromethane as the solvent.^95^ All electronic structure calculations were performed using Gaussian16.^96^

### Why Start with the Pure PBE Functional?

PBE employs the Generalized Gradient Approximation (GGA) and does not include any HF exchange.^97^ It is therefore a pure functional that belongs to the second rung of the “Jacob’s ladder” of functionals.^93^ Instead, PBE0 (also known as PBE1PBE) belongs to the fourth rung of the ladder and is classified among the class of hybrid GGA by incorporating 25% HF exchange and 75% DFT exchange.^98^

Leveling up in Jacob’s ladder of DFT functionals involves using more sophisticated methods and is therefore generally expected to provide more accurate energies. In fact, PBE0 is a popular choice for organic materials and for many small transition metal complexes with a single metal center or a small number of metal centers.^99–105^ However, the pure PBE functional is a popular choice for certain classes of gold^21,89,90,92,106–112^ and copper nanoclusters.^113–116^ The use of pure functionals in those clusters was motivated by their strong agreement, on multiple occasions, with experimentally determined band gaps. For example, Zeng et al.^117^ computed an optical gap in the range of 2.14–2.28 eV for Au_20_(SR)_16_ (R = CH_2_CH_2_PH), which has a closely related gold core structure to the Au_20_(TBBT)_16_ cluster investigated in this work. Their computed optical gap is in excellent agreement with the 2.15 eV optical gap measured experimentally by Jin and co-workers.^107,108^

### Testing the Effect of HF Exchange and Range-Separation Parameter

While the use of PBE has been primarily justified by agreement with experiments, there appear to be relatively few benchmark studies that investigate the effect of moving up Jacob’s ladder to hybrid functionals in these systems.^118^

Hybrid functionals introduce some percentage of HF exchange to the functional. We therefore customized PBE by varying amounts of HF exchange ranging from 0% (i.e., PBE) to 25% (PBE0) and beyond to 50% (corresponding to PBE50) in increments of 5%.

Next, we tested the effect of using a long-range separated hybrid (LC-ωPBE) and tuned the range separation parameter (ω) from 0.001 to 0.5 bohr^–1^ (0.001 < ω < 0.5 *a*_0_^–1^ where *a*_0_^–1^ denotes the Bohr radius). LC-ωPBE by default includes 0% HF exchange at short-range (SR), 100% HF exchange at long-range (LR) and an ω of 0.4 bohr^–1^.^119^ We also test a second approach where the SR exchange is described using 20% HF and 80% PBE while the LR exchange is set to 100% HF, in the style of LRC-ωPBEh.^120^ Following ref 120. we use the default ω = 0.2 bohr^–1^, but then tuned the range separation parameter 0.001 < ω < 0.5 *a*_0_^–1^. For additional information on how this tuning was done, see Supporting Information S2.1, S2.2, and S2.3.

### Frontier Molecular Orbital Analysis

Frontier orbital (HOMO and LUMO) analysis was carried out by determining the percent contribution of different basis functions (metal *s* and *p* functions, metal *d* functions, and each ligand type) to each molecular orbital.

### Testing the Correlation Functional and Basis Set

HOMO–LUMO energy gaps were computed for Cu_14_ and Au_20_ using eight different pure functionals (XAlpha, SVWN5, SOGGA11, PBE, BP86, TPSSTPSS, MN15L and OLYP) and five different basis sets (def2-SV­(P), def2-SVP, def2-SVPD, def2-QZVP, and def2-TZVP).

### Energy Gap Calculations

VIEs (corresponding to X → *X* ^+^ + *e* ^–^) were calculated using the ΔSCF approach by taking the difference between the DFT energy of *X* ^+^ and the DFT energy of *X* both computed at the equilibrium geometry of *X*. Similarly, VEAs (X + *e* ^–^→ *X* ^–^) are energy differences between *X* ^–^ and *X* computed at the equilibrium geometry of *X*. VEEs were calculated using a single point TD-DFT calculation at the optimized geometry of the ground state.

For VEE, VIE, and VEA, nonequilibrium solvation was used (ne-PCM). However, we also calculated (V)­EE, (V)­IE and (V)­EA energies using equilibrium e-PCM for benchmarking purposes. As indicated earlier, the use of vertical energies with equilibrium solvation is simply a theoretical construct that has no experimental counterpart, but is convenient because it includes a major ingredient (the solvent relaxation) of an equilibrium process without the additional computational cost of geometry optimization and frequency calculations needed for equilibrium calculations. In Markus theory terms, the solvent relaxation is the outer sphere relaxation energy (λ_*o*_) and the nanocluster geometry rearrangement is the inner sphere relaxation energy (λ_*i*_). Since λ_*o*_ ≫ λ_*i*_, it is reasonable to just use λ_*o*_ to estimate the total reorganization energy. Gas-phase calculations were performed for both clusters to serve as a reference for solvation effects on VIE-VEA, HOMO–LUMO, or TD-DFT energy gaps. For more information on how this was done, see Supporting Information S2.4 and S2.5.

Reduction and oxidation potentials were calculated with varying % HF only for the truncated [Cu_14_H_10_(MBN)_3_(PH_3_)_8_]^+^ model by optimizing the geometries of the open-shell cationic *X* ^+^ and anionic *X* ^–^ species and running frequency calculations at the optimized geometry to confirm all positive frequencies. Thermodynamic quantities from the frequency calculations obtained for the *X*, *X* ^+^ and *X* ^–^ systems were used to compute the relative free energies at a temperature of 298.15 K and pressure of 1 atm using unscaled frequency values. Redox potentials were calculated using eqs 2 and 3.

For Au_20_, the size of the cluster prevented us from computing *E*°_ox_ – *E*°_red_ so we instead compute the more approximate (V)­IE-(V)­EA energy gap.

## Results and Discussion

### [Cu_14_H_10_(MBN)_3_(PH_3_)_8_]^+^

#### How Different Energy Gaps Compare to the Experimental Electrochemical Gap

Table 1 presents some “band gap” calculations carried out for different methods and continuum solvation models. Rows 1, 2, 4, and 6 reflect the four methods outlined in Figure 1. Those are referenced against the experimentally determined electrochemical gap provided at the bottom of the table. Based on the discussion presented in the introduction, we should expect that *E*°_ox_ – *E*°_red_ calculations should provide the best agreement with this band gap. Indeed, the energy reported at the def2-SVP level of theory and truncated model is in reasonably good agreement, at 2.29 eV (see row 6 in Table 1).

**Table 1:** Band Gap Values Computed with the Pure PBE Functional for Different Methods (HOMO-LUMO, VEE, VIE-VEA, and *E*°_ox_ – *E*°_red_) and Different Solvation Models (e-PCM and ne-PCM)^a^

row	method	solvation	energy (eV)
1	HOMO–LUMO	ne-PCM	1.84
2	VIE-VEA	ne-PCM	3.33
3	(V)IE-(V)EA	e-PCM	2.54
4	VEE	ne-PCM	1.85
5	(V)EE	e-PCM	1.85
6	*E*°_ox_ – *E*°_red_	e-PCM	2.29
experimental electrochemical band gap			**2.07**_3_

a All calculations are carried out for the truncated [Cu_14_H_10_(MBN)_3_(PH_3_)_8_]^+^ model.

Two other approximations, the HOMO–LUMO gap and VEE computed with TD-DFT, are in the range of 1.84–1.85 eV, in good agreement with each other and with the electrochemical band gap. On the other hand, the computed fundamental gap, representing a vertical VIE-VEA gap, is much larger, at 3.33 eV. This difference between the fundamental gap and other energy gaps can mainly be attributed to the sensitivity of the fundamental gap to solvent effects; simply relaxing the solvent using the equilibrium formalism of PCM (e-PCM) yields back an energy (2.54 eV) that is closer to *E*°_ox_ – *E*°_red_. Solvent relaxation alone (from ne-PCM to e-PCM) therefore reduces the fundamental gap by close to 0.8 eV, corresponding approximately to the outer sphere reorganization energy λ_*o*_.^121^ In comparison, moving from (V)­IE-(V)­EA to *E*°_ox_ – *E*°_red_ only modifies the energy by 0.25 eV, which largely can be attributed to geometry relaxation (i.e., inner sphere reorganization λ_*i*_) and thermal corrections. Therefore, as alluded to earlier, solvent relaxation plays a more important role in reproducing the electrochemical band gap than accounting for geometry and thermal corrections. Notice that solvent relaxation has a much smaller (even negligible) effect on VEE; the excitation energy computed using e-PCM and ne-PCM are identical to the second decimal place.

The best approximation to the band gap, *E*°_ox_ – *E*°_red_, still overestimates the band gap by a little over 0.2 eV. However, with the level of approximations employed here (and typically across the entire nanocluster field), caution needs to be exercised in making claims about accuracies on the order of 0.1–0.2 eV or smaller. Such errors can easily be introduced due to the use of a finite basis set, truncated ligand model, ignoring relativistic/spin–orbit coupling effects,^122–125^ and/or by using the vertical approximation (in the case of VEE or VIE-VEA). Moreover, calculations represent the complex environment near the electrode by assuming a homogeneous polarizable continuum. In reality, a typical electrochemical setup has the nanoclusters surrounded by solution ions that form a double layer near the electrode surface. The measured redox potential of the nanocluster is therefore associated not only with an intrinsic change in its redox state but also includes contributions from electrostatic interactions between the transferred electron and the counterion in the surrounding environment, as well as an environment of varying dielectrics involving the metal core, ligands, electrolyte ions and solvent. One approach to estimate the magnitude of such effects experimentally is to measure the charging energy, which is obtained from the redox potential of adding/removing an additional electron. The charging energy is sometimes subtracted from the “measured” electrochemical gap to estimate a true “intrinsic” band gap of the system. In Cu_14_, this energy separation between the two peaks appears to be 0.33_8_ eV, while in Au_20_, it is 0.20 eV. However, the charging energy includes not only the electron’s screened interactions with the surrounding double-layer environment, but also the Coulombic repulsion of the newly added electron/hole with one(s) already on the nanocluster.^126^ Therefore, it remains difficult to isolate the solvent’s effect on screening the interactions between the nanocluster with the double layer from its effect on directly stabilizing the reduced/oxidized nanocluster. In any case, the error associated with using a homogeneous environment in the calculations in place of the electrode environment is likely to be relatively small compared to other sources of error. This can be generally observed in the literature, where studies find a reasonably good agreement with measured electrochemical band gaps regardless of whether they subtract the charging energy or not.

The relatively good agreement between HOMO–LUMO gap, (V)­IE-(V)­EA, VEE, (V)­EE, and *E*°_ox_ – *E*°_red_, spanning a range of 1.84–2.54 eV, appears at first glance encouraging and indicates that it is possible to use any one of those quantities to represent a system’s band gap. However, as discussed later in this section, changing the electronic structure method (e.g., by increasing the % HF exchange) differentially affects these band gaps.

#### The Effect of Truncating the Inert Triphenylphosphine Ligands

Throughout most of this work, we use the truncated [Cu_14_H_10_(MBN)_3_(PH_3_)_8_]^+^ model where the triphenylphosphine PPh_3_ ligands are replaced with phosphine PH_3_. To check whether the truncated model is suitable for the purpose of this benchmark, we computed HOMO–LUMO gaps and TD-DFT energies for both the full and truncated models as a function of varying % HF exchange. The full model consistently predicts a slightly lower HOMO–LUMO gap and TD-DFT energies than the truncated model, but this effect is on the order of 0.1 eV in solution across the full range of % HF exchange tested (from 0 to 50%). In gas-phase, the full model also underestimates the HOMO–LUMO gap and TD-DFT energies compared to the truncated model on the order of 0.2 eV across the full range of % HF exchange, a slightly higher difference compared to the solvated model (see Figures S2 and S3, Tables S2, S3, S4, and S6). Therefore, the effect of using truncated model on the energy of the system is relatively small regardless whether using a solvated or a gas-phase model.^127^ Importantly, this stabilization is consistent across all functionals with varying % HF exchange, indicating that the full and truncated models behave similarly across both pure and hybrid PBE functionals.

Later in this section, we show orbital analyses and NTOs computed using different functionals for both the full and truncated Cu_14_ models. Those analyses will further confirm that the PPh_3_ or PH_3_ do not contribute to the frontier (HOMO–LUMO or NTO electron–hole) orbital pairs. However, the presence of PPh_3_ can affect the shape of the orbitals through small modulations of the symmetry of the nanocluster or the charge-transfer character of the excitation.

#### Sensitivity of the Four Computed Energy Gaps to HF Exchange

Figure 4a displays the change in the four computational “band gap” approaches (HOMO–LUMO, fundamental, optical, and electrochemical) as a function of % HF introduced to the PBE functional. We find that the results obtained with the pure PBE functional can change dramatically when moving to hybrid functionals with varying degrees of HF exchange. None of the energy gap vs HF exchange plots are parallel to each other. The HOMO–LUMO gap exhibits by far the highest sensitivity to % HF exchange, ranging from 1.84 eV at 0% HF to 5.74 eV at 50% HF, more than tripling in magnitude in this range. The other energy gaps also increase significantly with the introduction of HF exchange, but this increase is more muted than for HOMO–LUMO. *E*°_ox_ – *E*°_red_ shows the lowest degree of dependence, but still ranges from 2.29 eV at 0% HF to 3.75 eV at 50% HF, a range of 1.46 eV.

**Figure 4:**
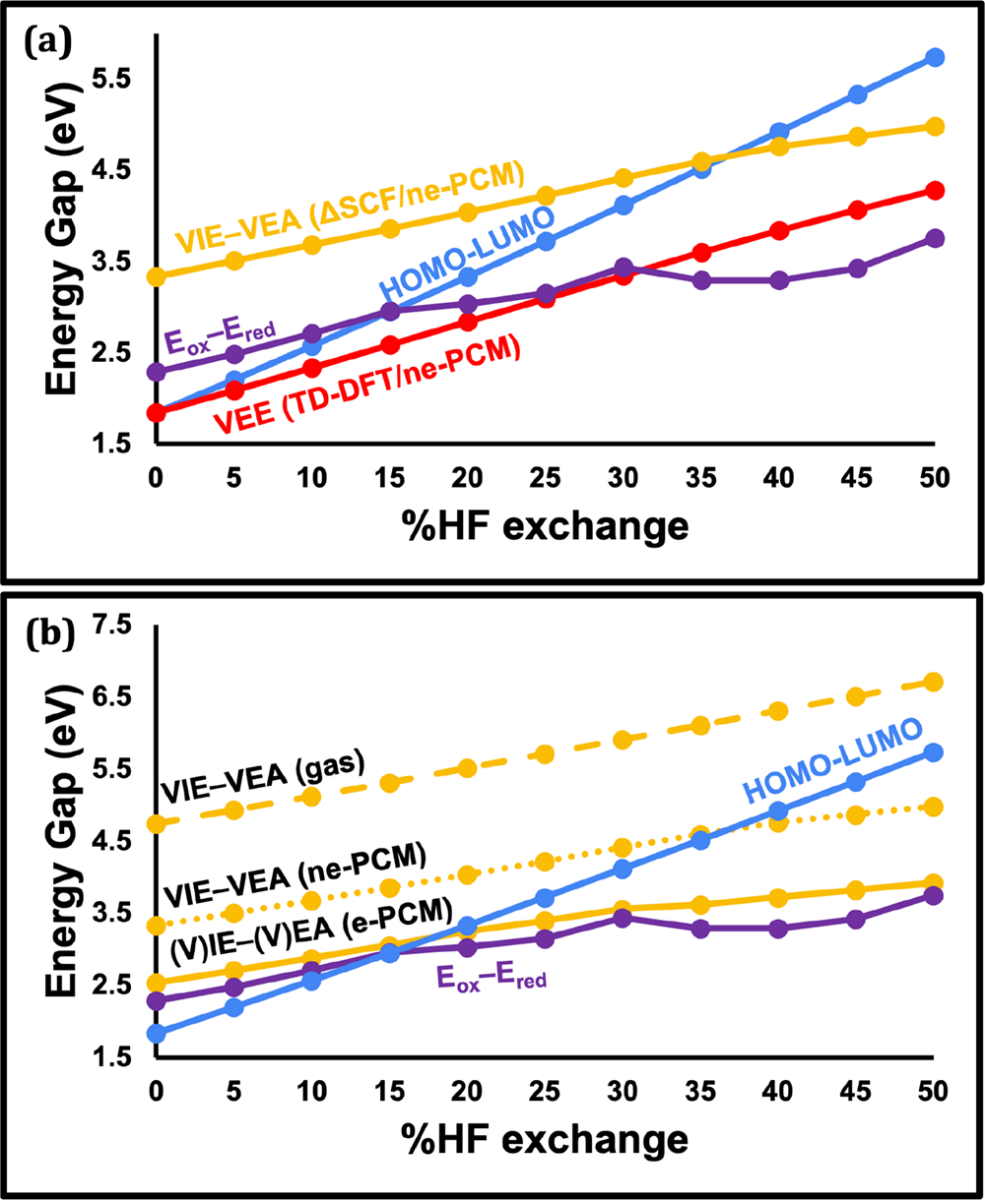
(a) HOMO–LUMO (blue line), VIE-VEA (yellow line), VEE (red-line) and *E*°_ox_ – *E*°_red_ (purple line) energy gaps for [Cu_14_H_10_(MBN)_3_(PH_3_)_8_]^+^ as a function of varying the HF exchange from 0 to 50% in the PBE functional. (b) The same VIE-VEA (now indicated using a dotted yellow line) repeated with equilibrium solvation (solid line) and without solvent; i.e., gas-phase (dashed line). The HOMO–LUMO (blue line) and *E*°_ox_ – *E*°_red_ (purple line) plots from panel (a) are kept to serve as a reference. See SI Table S4 for energies plotted in panel (a) and SI Table S5 for the energies plotted in panel (b).

Due to the nonparallelity of the different energy gaps as a function of HF exchange, the energetic ordering of those different gaps changes qualitatively for different functionals. At 0% HF, the VEE and HOMO–LUMO gaps agree well with each other and are smaller than the *E*°_ox_ – *E*°_red_ and VIE-VEA energy gaps. At 25% HF, corresponding to PBE0, the optical and electrochemical gaps become almost identical and are lower in energy than the HOMO–LUMO gap and VIE-VEA. At 50% HF, the order again switches with *E*°_ox_ – *E*°_red_ giving the lowest energy gap, followed by VEE, VIE-VEA, and HOMO–LUMO in energetic order.

#### The Effect of Solvation on VIE-VEA vs VEE

Figure 4b compares VIE-VEA calculations carried out in the gas phase to those computed using ne-PCM and e-PCM. Equilibrium solvation reduces the VIE-VEA gap by almost 2.2 eV compared to the gas phase. The magnitude of the solvation on VIE-VEA is not surprising when considering that VIE and VEA energies are computed from states with different charge (in the case of Cu_14_, +1 charge for the initial state, +2 for the ionized/oxidized state, and 0 for the electron-attached/reduced state). The VIE-VEA energies with ne-PCM are almost 1 eV higher in energy than the e-PCM calculations. The (V)­IE-(V)­EA and *E*°_ox_ – *E*°_red_ lines in Figure 4b are almost parallel, with a separation ranging from 0.1 to 0.43 eV (depending on the % HF exchange used). Therefore, the (V)­IE-(V)­EA approach serves as a useful and computationally efficient approximation to the more time-consuming *E*°_ox_ – *E*°_red_ calculations.

As shown in SI Figure S4, in contrast to the VIE-VEA calculations, TD-DFT VEEs are much less sensitive to the details of the solvent model, despite the S_1_ state having MLCT character. VEE­(ne-PCM) and VEE­(e-PCM) plots can barely be distinguished from each other in that figure.

The smaller slope of the VEE, VIE-VEA, and *E*°_ox_ – *E*°_red_ lines relative to HOMO–LUMO in Figure 4a can be simply understood through the variational principle; these three methods include an extra degree of relaxation of the wave function compared to orbital energies. That is, the wave function is further optimized after accounting for an electronic excitation or ionization/attachment. Therefore, they have higher flexibility to approach the optimal solution of the wave function compared to orbital energy solutions. However, they are still limited by the quality of the functional; an inaccurate functional would converge to incorrect TD-DFT, VIE-VEA, and *E*°_ox_ – *E*°_red_ energies.

As mentioned in the Theoretical Background section, the quality of a functional may be probed by the agreement between the HOMO–LUMO and the fundamental energy gap (VIE-VEA) in view of the extended Koopmans’ theorem. However, solvation effects complicate this comparison. The HOMO–LUMO gap does not intersect with the VIE-VEA line anywhere between 0 and 50% HF exchange in the gas phase, but it intersects at near 35% HF exchange with VIE-VEA­(ne-PCM) and at around 15–20% HF exchange for VIE-VEA­(e-PCM). Meanwhile, although HOMO and LUMO absolute orbital energies are affected by solvation, the HOMO–LUMO gap is relatively insensitive to solvation (this will become clear when comparing panels (a) and (b) of Figure 5). Therefore, tuning the HF exchange (or the long-range parameter for range-separated functionals) will yield different optimal parameters if the calculations are done with vs without solvent effects. In this case, it is clear that neither the gas phase nor solvent-phase VIE-VEA calculations give the optimal functional that agrees best with the experiment; the pure PBE functional (0% HF exchange) instead appears to give the best match with experiments.

**Figure 5:**
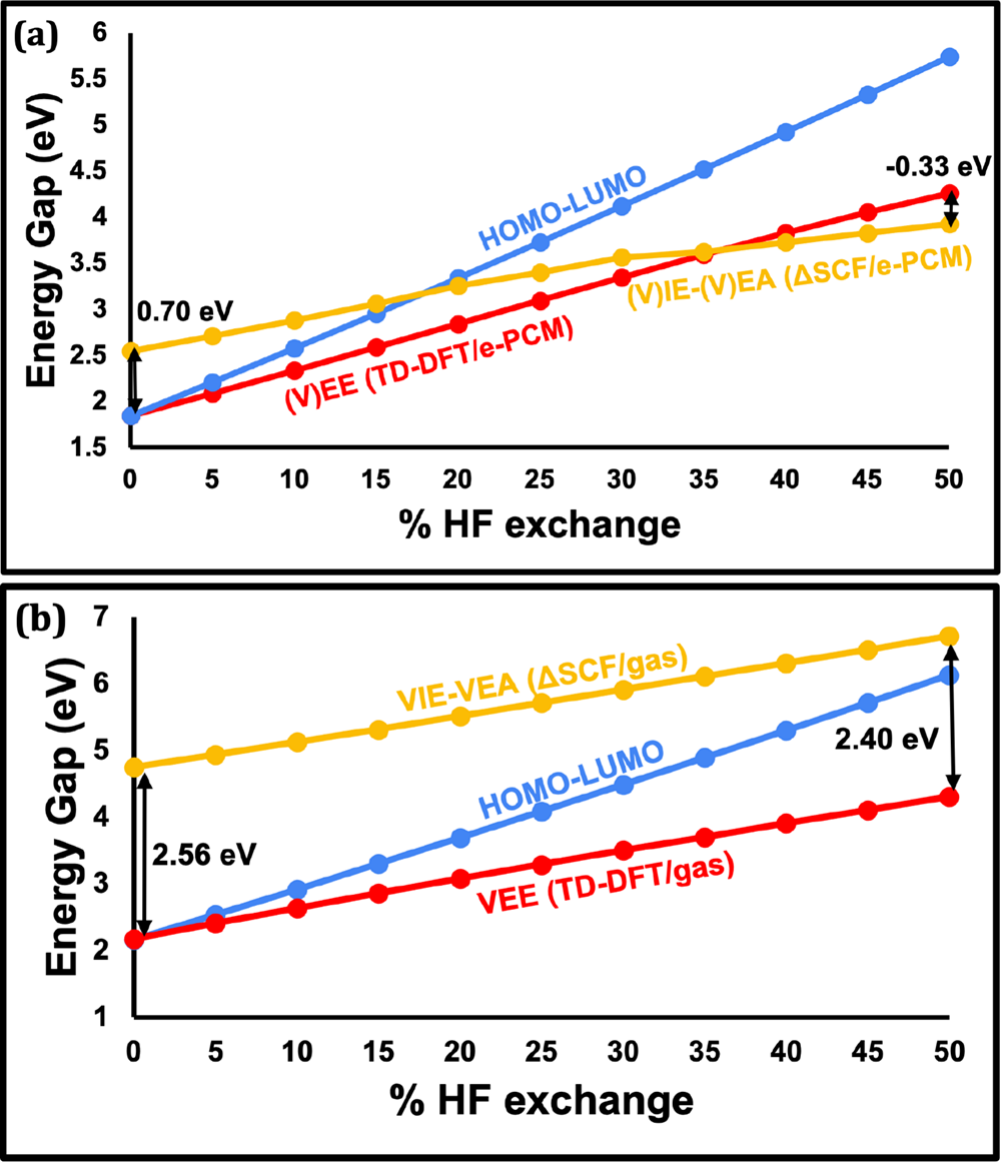
(a) A comparison of HOMO–LUMO (blue line), (V)­IE-(V)­EA (yellow line) and (V)­EE (red-line) calculations for [Cu_14_H_10_(MBN)_3_(PH_3_)_8_]^+^, all computed with equilibrium PCM. The difference between (V)­IE-(V)­EA and (V)­EE computed with TD-DFT is indicated by black arrows at 0% HF and 50% HF corresponding to 0.70 and −0.33 eV, respectively. (b) The same calculations carried out in the gas-phase. The difference between VIE-VEA and TD-DFT indicated here is 2.56 eV at 0% HF and 2.40 eV at 50% HF. See SI Tables S4–S6 for the energies plotted in this figure.

For a better comparison of calculations carried out in solution vs gas phase, Figure 5 plots TD-DFT, (V)­IE-(V)­EA, and HOMO–LUMO gap energies computed with e-PCM (Figure 5a) and gas phase (Figure 5b). In both the gas phase and in solution, VEEs appear to intersect with the HOMO–LUMO line at 0% HF. Since the VEE is considerably less sensitive to solvation effects than VIE-VEA, it appears here to be a more useful reference to compare the HOMO–LUMO gap to. This would mark a deviation from using Koopmans’ theorem (i.e., using VIE-VEA), but for solvated molecules may still be useful to obtain energies within a reasonable error relative to the experiments.^83^ This discussion will be revisited in the Conclusion section after presenting the results of the Au_20_ cluster.

#### Changing Solvation Energies As a Function of HF Exchange

Another observation from Figure 5a is that (V)­EE energies are not parallel to the (V)­IE-(V)­EA energies. The double-headed arrows in Figure 5a,b highlight the difference in energy between the VEE and VIE-VEA lines at 0% HF and 50% HF. While this difference is relatively constant in the gas phase (2.56 eV at 0% and 2.40 eV at 50% HF exchange, changing only by 0.16 eV), the (V)­EE and (V)­IE-(V)­EA energies in solution change from 0.70 eV at 0% HF to −0.33 eV at 50% HF, a difference of 1.06 eV. This loss of parallelity in Cu_14_ can therefore be attributed to the effect of solvation. This also can be observed in Figure S4, where the gas-phase TD-DFT calculations are not parallel to the TD-DFT/e-PCM and ne-PCM calculations. As discussed next, this variation in solvation energy as a function of % HF exchange can be explained by a change in the degree of the charge-transfer character of Cu_14_’s first excited state.

First, Figure 6 shows the percent contribution of atomic orbitals from different groups to the canonical HOMO and LUMO orbitals. Specifically, we categorize the orbitals as either coming from the copper metal *s/p* orbitals, Cu *d* orbitals, the 10 hydrides in the nanocluster core, the sulfur atom of the MBN ligand, the PH_3_ ligands, and the aromatic group in the MBN ligand. We find that the HOMO is dominated by the Cu­(d) orbitals while the LUMO is dominated by the MBN orbitals (see Figure 6a,b), consistent with an MLCT character. This charge transfer character is mostly stable to changes in % HF exchange, with only subtle variations as a part of the HOMO becomes localized on the hydride instead of the copper. Such subtle changes in electronic character do not immediately explain the difference in solvation energy at low vs high HF exchange. However, it is worth remembering here that canonical molecular orbitals are associated to ionization, rather than excitation. In fact, the gas, ne-PCM, and e-PCM VIE-VEA energies are largely parallel to each other (see Figure 4b), consistent with a mostly stable orbital character with changing HF exchange. To understand the dependence of VEE on solvation, it is more suitable to look at the one-photon NTOs, which are presented in Figure 7.

**Figure 6:**
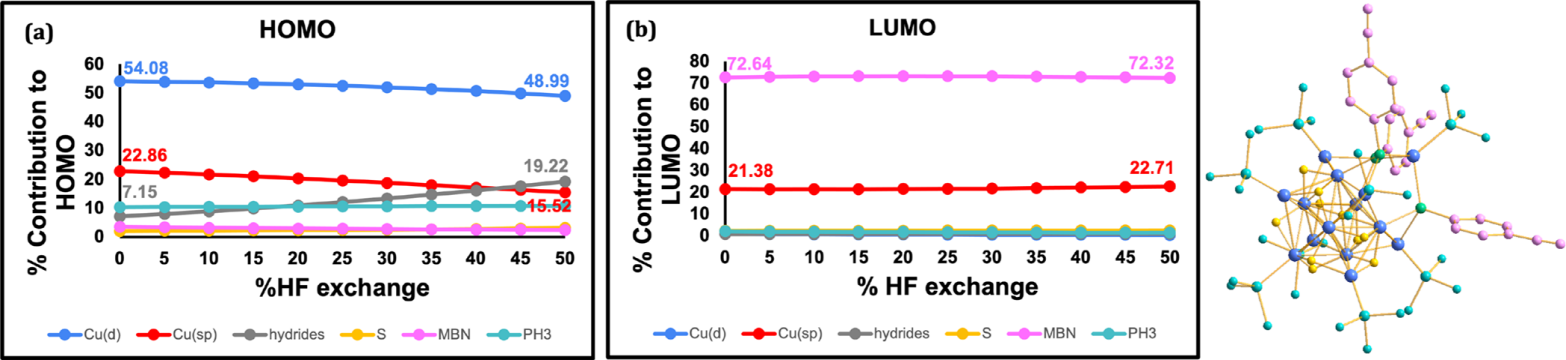
HOMO and LUMO orbital analyses for [Cu_14_H_10_(MBN)_3_(PH_3_)_8_]^+^ as a function of % HF incorporated into the PBE functional. (a) The contributions of Cu­(d) (blue), Cu­(sp) (red), the 10 hydrides (gray), S (yellow), MBN (pink), and PH_3_ orbitals (cyan) to the HOMO. (b) The contribution of the same atomic orbitals to the LUMO. The structure on the right shows the truncated Cu_14_ structure with the atoms colored according to the orbital categories used (blue is used for copper since both red and blue are used in the plot).

**Figure 7:**
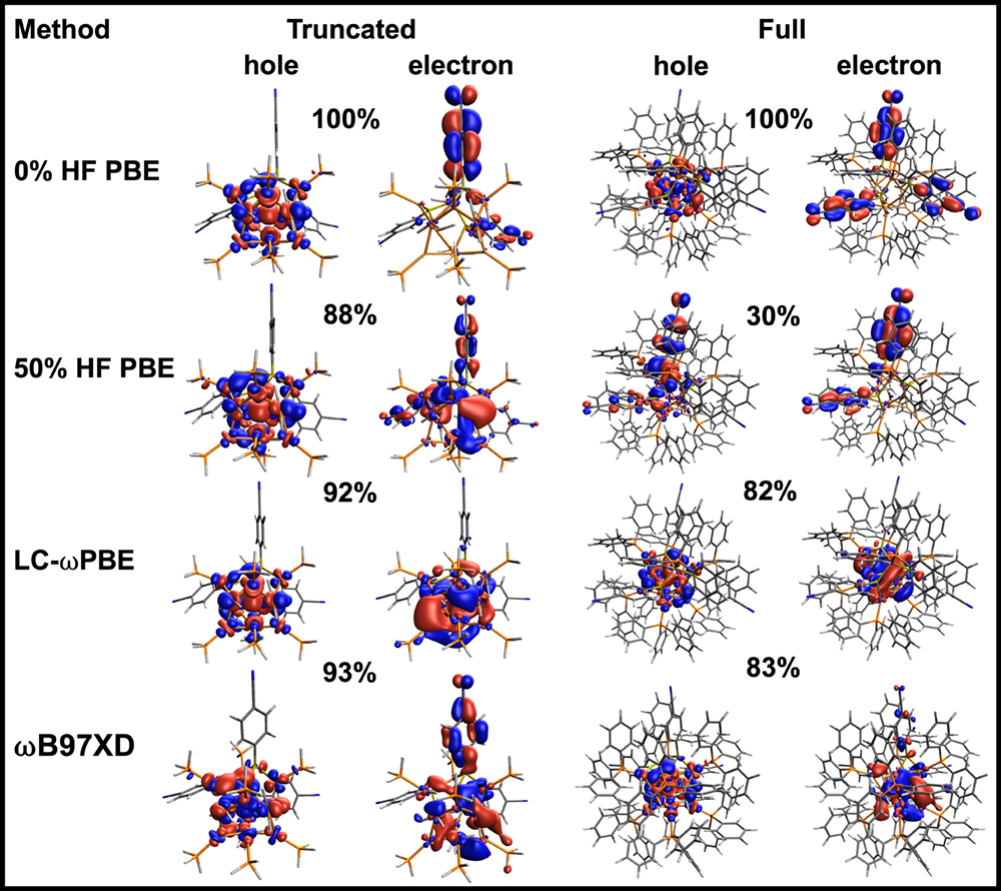
Cu_14_ truncated and full models isocontour representation (isovalue 0.07 au) of the NTOs (hole: left; electron: right) associated with the first excited state of Cu_14_ computed at the PBE, LC-wPBE, 50% HF PBE or PBE50 and ωB97XD levels. The percent contribution of the electron–hole pair to the full transition is also indicated for each orbital pair.

Figure 7 presents the NTOs corresponding to the first singlet excited state of Cu_14_ for both the pure functional (0% HF) and for the global hybrid with 50% HF. We also test the effect of using two long-range corrected functionals, LC-ωPBE and ωB97XD, which will be introduced in more detail later in the manuscript. The NTOs are presented for both the truncated and the full model for comparison. The pure PBE functional shows the strongest MLCT character, with the NTO hole ortibal fully localized on the copper hydride and the NTO electron ortibal almost fully localized on the MBN ligands. On the other hand, PBE50 NTOs show a weakening in the CT character both in the truncated and the full model. In the truncated model, the NTO electron orbital has some density on the metal core and a smaller density on the MBN ligand.

These changes in the NTOs explain the changes in solvation energy as a function of HF exchange and the resulting nonparallellity of the (V)­EE and (V)­IE-(V)­EA lines in Figure 5a. Specifically, while the canonical HOMO and LUMO maintain a relatively constant character as a function of HF exchange, one of the NTOs becomes more delocalized with increasing HF exchange, reducing the excited state dipole moment. At low % HF exchange (where the charge transfer is largest), a larger solvation energy results in a (V)­EE that is lower in energy than (V)­IE-(V)­EA. Increasing the HF exchange then results in a decreasing solvation energy and a steepening of the (V)­EE vs HF exchange slope.

As discussed later for the Au_20_ cluster, where the HOMO–LUMO and NTOs both consistently have metal-to-metal character and no charge transfer, the (V)­IE-(V)­EA and VEE plots as a function of % HF exchange remain parallel.

The NTOs computed using long-range corrected functionals in Figure 7 also show reduced CT character compared to PBE. In the case of LC-ωPBE, the excitation is fully localized on the metal core. ωB97XD is an intermediate case and shows some MLCT character still. The persisting MLCT character in PBE50 and ωB97XD provides further confidence that this system truly has CT character in its first excited state, in agreement with experimental observations showing that the CN stretch in MBN shifts downfield in the excited state.^19^

The calculations on the full model, which include the PPh_3_ instead of the PH_3_ ligands, also largely give the same conclusion about the CT character of the first singlet excited state. Irrespective of the model used (full/truncated), there is no electron/hole density on the phosphine ligands, which helps explain why using a truncated model gives similar band gap energies (and similar energy trends as a function of HF exchange) as the full model. However, the NTO shape in the full model and the truncated model have some differences, which can probably be attributed to changes in the degree of symmetry of the structures and/or to subtle electronic effects of the PPh_3_ ligand.

### Au_20_(TBBT)_16_

#### The Four Energy Gaps vs HF Exchange

To test whether the conclusions from the Cu_14_ system are transferable to another system, we investigate Au_20_(TBBT)_16_ (hereon, Au_20_), a thioprotected gold nanocluster. Unlike Cu_14_, Au_20_’s first excited state is optically active and has local metal-to-metal instead of charge transfer character.^117^

Due to the larger size of this system, *E*°_ox_ – *E*°_red_ calculations were replaced by the more approximate (V)­IE-(V)­EA/e-PCM calculations, which are expected to give a reasonable trend consistent with *E*°_ox_ – *E*°_red_ as illustrated for Cu_14_ in Figure 4b.

Figure 8 compares the HOMO–LUMO, VIE-VEA, (V)­IE-(V)­EA, and VEE calculations for Au_20_. Surprisingly, despite the very different electronic characters of Cu_14_ and Au_20_, Figure 8 closely resembles Figures 4a and 5a, with only one main difference; the (V)­IE-(V)­EA/e-PCM and VEE curves are parallel for Au_20_, maintaining a difference of 0.36 eV at 0% HF to 0.26 eV at 50% HF. Instead, those two lines cross in Cu_14_, i.e., the (V)­IE-(V)­EA/e-PCM gap becomes lower than the (V)­EE at high % HF exchange (recall here that VEE, computed with nonequilibrium solvation, was virtually indistinguishable from (V)­EE which is computed with equilibrium solvation in Cu_14_). Therefore, Au_20_ behaves consistent with expectations that the optical gap is slightly lower than the fundamental gap.^18^ As anticipated earlier and will be discussed in more detail below, this is because the HOMO–LUMO and NTO orbital character in Au_20_ are similar.

**Figure 8:**
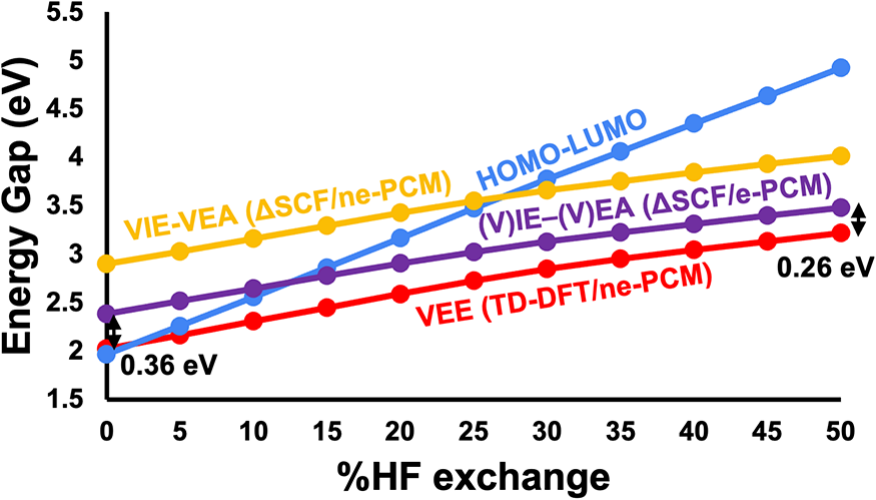
HOMO–LUMO (blue line), (V)­IE-(V)­EA (purple line), VIE-VEA (yellow line) and VEE (red line) energy gaps for a truncated Au_20_(SCH_3_)_16_ model as a function of varying the HF exchange from 0 to 50% in the PBE functional. The difference between (V)­IE-(V)­EA and TD-DFT is indicated by black arrows at 0% HF and 50% HF corresponding to 0.36 and 0.26 eV, respectively. See Table S7 for the energies plotted in this figure.

Before discussing the differences between Cu_14_ (Figures 4a and 5a) and Au_20_ (Figure 8), we start by highlighting the similarities. Consistently with Cu_14_, Au_20_’s HOMO–LUMO gap is very sensitive to the DFT functional (varying from 1.96 eV with 0% HF to 4.92 eV with 50% HF, a 2.5-fold difference). VIE-VEA, (V)­IE-(V)­EA and VEE are less sensitive to HF exchange. Again, this is because these methods account for the response of the electrons to excitation, ionization, and electron attachment.

#### Comparison to the Experimentally Measured Optical and Electrochemical Energy Gap

Figure 8 shows that the HOMO–LUMO gap matches with the VEE only for low (0–5%) HF exchange, similar to what was found for Cu_14_. Those 0–5% HF exchange calculations also match well with the experimental 2.12 eV optical band gap obtained from the UV–vis absorbance spectra shown in Figure 3d. Specifically, at 0% HF, the TD-DFT energy gap is 2.02 eV and at 5% HF it is 2.16 eV. Further increasing the % HF exchange overestimates the optical gap. This may explain why most studies on Au nanoclusters in recent years have used pure PBE instead of PBE0 for optical band gap predictions, giving a good agreement with experimental data (see for instance refs 21,89,90,92,106–108,110,111 ).

The electrochemical band gap measured in this work for Au_20_ is 1.82_6_ eV (see Figure 3c). Chen et al. electrochemically measured an oxidation potential of 0.74 V and a reduction potential of −1.13 V for the same nanocluster, giving an electrochemical band gap of 1.87 eV, consistent with our results.^111^ While our (V)­IE-(V)­EA/e-PCM value overestimates this experimental band gap (predicting a value of 2.38 eV at 0% HF with PBE/def2-SVP shown by the purple line in Figure 8 and reported in Table S7), this overestimation is consistent with the overestimation also observed for (V)­IE-(V)­EA Cu_14_ in row 3 of Table 1. A better agreement is expected if one computes the full *E*°_ox_ – *E*°_red_ energy, as done for Cu_14_, and increases the basis set size, as discussed below.

#### The Orbital Character for Au Results in Parallel VEE and VIE-VEA Lines vs HF Exchange

In Au_20_, the increase in VEE as a function of HF exchange is comparable to the increase in (V)­IE-(V)­EA/e-PCM, giving almost parallel lines. Specifically, the TD-DFT optical gap is consistently lower by around 0.36–0.26 eV at all % HF values. As discussed in the Theoretical Background section, VIE-VEA and VEE are fundamentally different quantities because the electron affinity involves an additional Coulombic electron–electron repulsion, often resulting in a fundamental gap that is higher than the optical gap. Au_20_ therefore behaves as expected, unlike Cu_14_ where the VEE becomes higher in energy than the (V)­IE-(V)­EA at functionals that have 35% HF exchange or more (Figure 5a). In Cu_14_, the lack of parallelity was explained by the evolution of the NTO orbital character as a function of HF exchange; an increase in % HF exchange led to a decrease in charge transfer character in the excited state while maintaining a strong CT character in the canonical HOMO and LUMO orbitals that are better connected with VIE-VEA. The corresponding canonical HOMO and LUMO character plots for Au_20_ are shown in Figure 9a,b, while the NTOs for the first excited state are shown in Figure 10.

**Figure 9:**
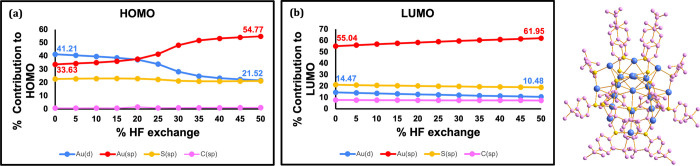
HOMO and LUMO orbital analyses for Au_20_ as a function of % HF incorporated into the PBE functional. (a) The contributions of Au­(d) (blue), Au­(sp) (red), S­(sp) (yellow), and C­(sp) (pink) to the HOMO. (b) Contribution of the same atomic orbitals to the LUMO. For each of the HOMO and LUMO plots, the % contribution of Au­(d) and Au­(sp) are shown in blue and red respectively. The structure on the right shows the truncated Au_20_(SCH_3_)_16_ structure with the atoms colored according to the orbital categories used (blue is used for gold since both red and blue are used in the plot).

**Figure 10:**
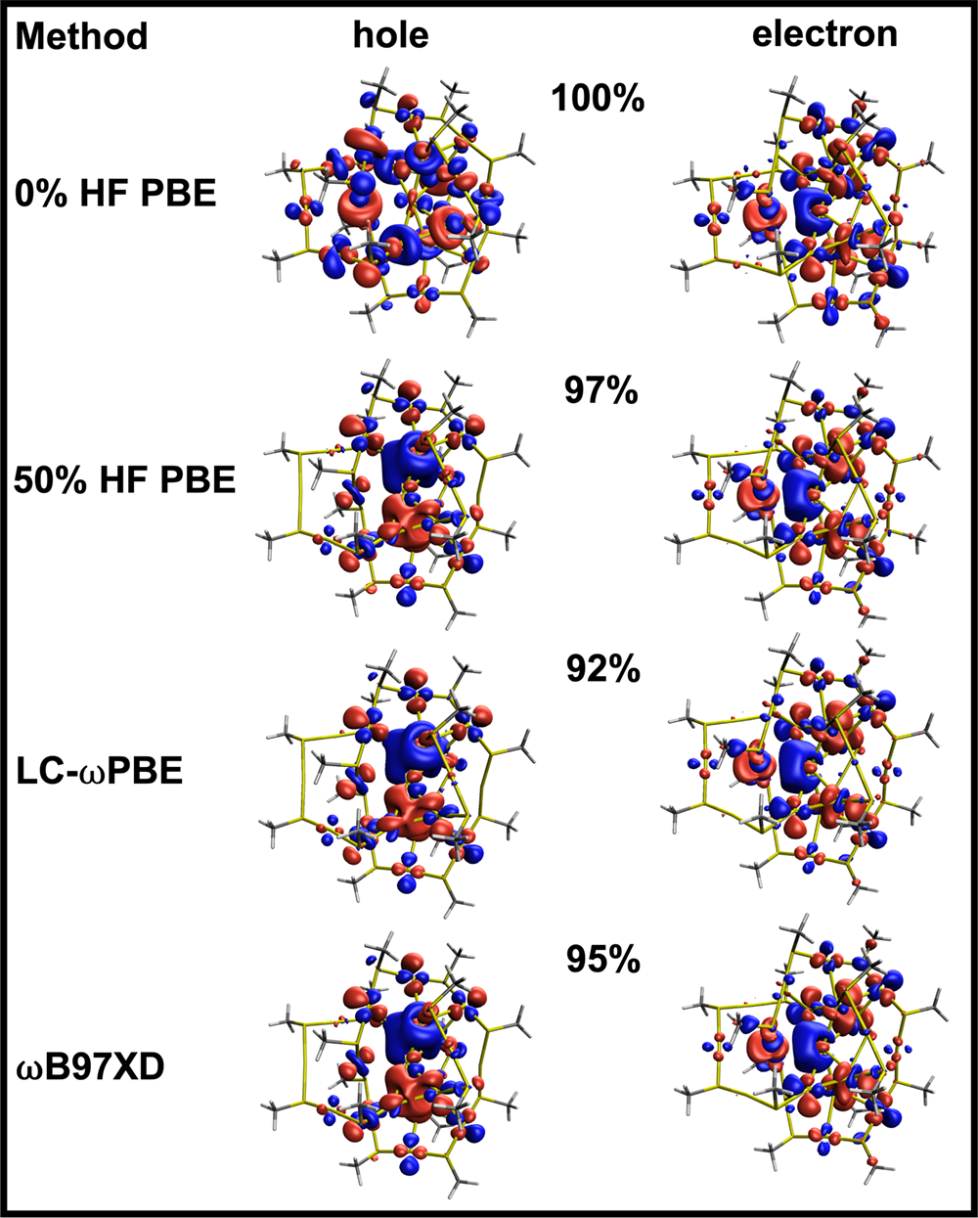
Au_20_ isocontour representation (isovalue 0.07 au) of the NTOs (hole: left; electron: right) associated with the first ES of Au_20_ computed at the PBE, LC-ωPBE, 50% HF PBE or PBE50 and ωB97XD levels. The percent contribution of the electron–hole pair to the full transition is also indicated for each orbital pair.

Orbital contributions in Figure 9a,b are assigned as coming from either Au­(d), Au­(sp), S­(sp) or C­(sp). Here, there appear to be more pronounced changes in orbital character than in Cu_14_ as a function of HF exchange, but those changes are associated exclusively with the Au orbitals (*d* vs *sp* character) and do not involve a change in the extent of charge transfer to the ligand. The NTOs (Figure 10) also appear to indicate an increasing mixing of *s* and *d* orbitals with increasing HF exchange, consistent with the canonical HOMO and LUMO molecular orbitals. However, both the canonical HOMO–LUMO and NTO orbitals show no charge transfer. The NTOs maintain their localized character on the Au core when using 0% HF exchange, 50% HF exchange or even long-range corrected functionals. As a result, there is no differential effect of the solvent on the TD-DFT and (V)­IE-(V)­EA energies, maintaining an optical gap lower than the fundamental gap.

The change from *d* to *sp* character in Au, a sixth row element, is due to the sensitivity of the *s* and *d* orbital energies to shielding from inner electrons.^128^ Changes in % HF exchange can have subtle effects on the relative energetics of those two orbitals. While such changes do not affect the metal-centered nature of the excitation, they may affect other properties.

### The Effect of Other Computational Parameters on HOMO–LUMO and Other Energy Gaps in Cu_14_ and Au_20_

#### The Effect of Range-Separated Functionals

Hybrid functionals attempt to resolve self-interaction error by introducing a certain percentage of HF exchange.^129^ However, range-separated hybrids are more modern functionals that are being used because they correctly capture the asymptotic long-range behavior of standard pure and hybrid functionals. Range-separated hybrids typically combine HF exchange at long-range (LR) with DFT exchange at short-range (SR).^71,130^ The distance at which this switch occurs is determined by the range-separation parameter, ω. In this work we test LC-ωPBE, which uses 100% PBE exchange at short-range and 100% HF exchange at long-range. We also tune LC-ωPBE to use 20% HF and 80% PBE SR exchange and 100% LR HF exchange in the style of LRC-ωPBEh.^120^ The results of tuning ω from 0.001 to 0.5 bohr^–1^ are reported in Figure 11. When ω is set to 0.001 bohr^–1^ (corresponding to introducing HF exchange at large distances, i.e, 1000 bohr), the HOMO–LUMO gaps converge toward the pure PBE functional for LC-ωPBE and to PBE20 for LRC-ωPBEh. On the other hand, with a larger ω value of 0.5 bohr^–1^, where the switch from DFT to HF exchange occurs at short distances of 2 bohr, the HOMO–LUMO gaps increase to around 7–9 eV. In the case of LC-ωPBE, this is an increase of almost 4-fold relative to the HOMO–LUMO gap computed with the pure functional.

**Figure 11:**
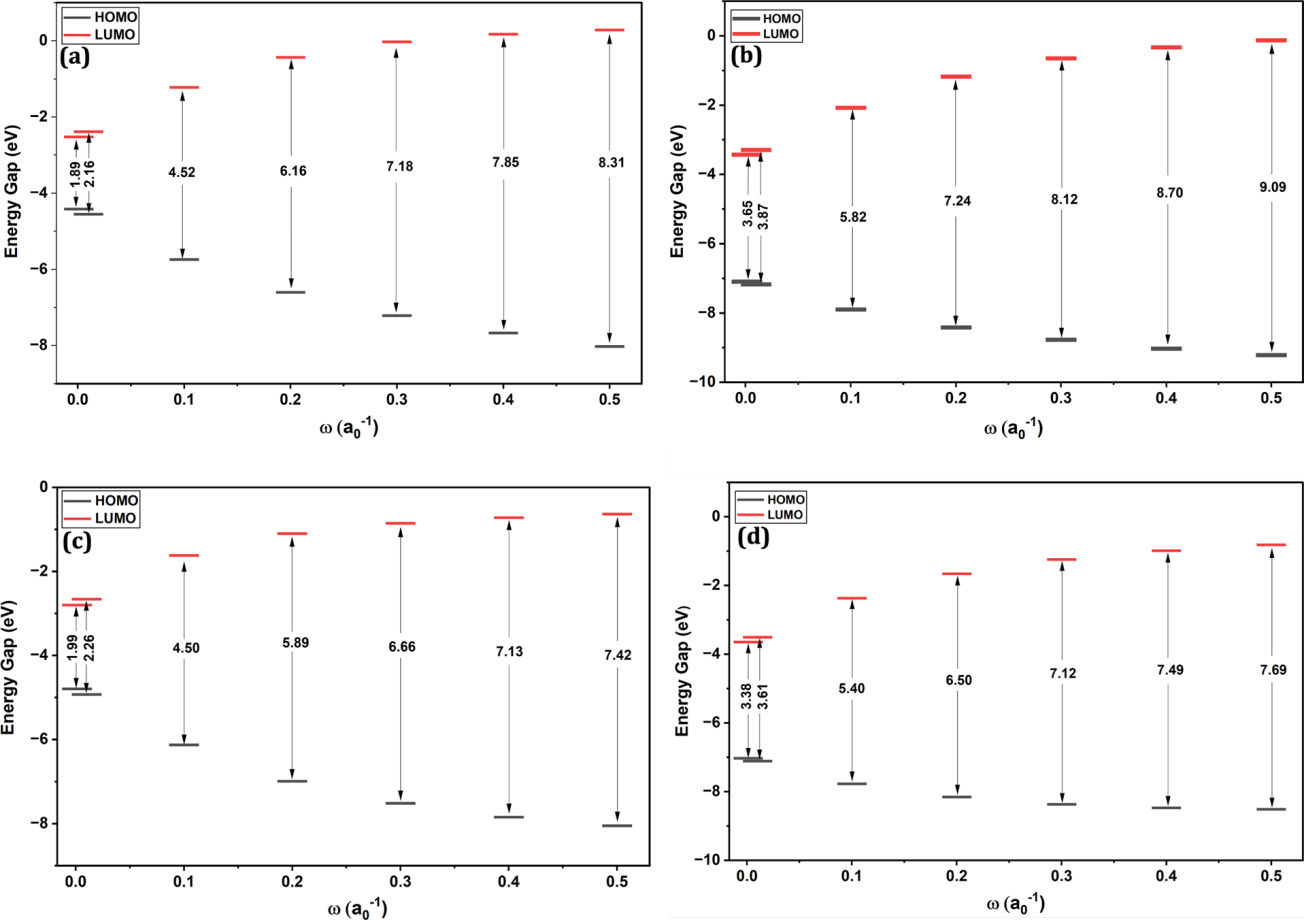
Effect of tuning ω in long-range corrected PBE on HOMO–LUMO energy gap. (a) truncated Cu_14_ HOMO–LUMO energy gap computed with LC-ωPBE (0% HF exchange at SR and 100% HF exchange at LR) and (b) the same LC-ωPBE functional tuned with 20% HF exchange at SR and 100% HF exchange at LR. (c,d) Au_20_ HOMO–LUMO energy gap computed at the same level of theory as Cu_14_ in panels a,b. See Table S9 for the energies plotted in panels (a) and (b), and Table S10 for the energies plotted in panels (c) and (d), respectively.

Just like HOMO–LUMO energy gaps were sensitive to global HF exchange, they are also very sensitive to the introduction of LR HF exchange. The long-range separated hybrids tested here lead to a rapid increase in HOMO–LUMO gap even at ω = 0.1 bohr^–1^. Using a larger value of ω will certainly lead to a significant overestimation of the computed optical, fundamental, and electrochemical energy gaps. For instance, as shown in Tables S9 and S10, The TD-DFT VEE computed with LC-ωPBE is 1.86 eV at ω = 0.001 bohr^–1^ and 4.18 eV at the default value of ω = 0.4 bohr^–1^, an increase of 2.32 eV). This is again consistent with the popularity of the pure PBE functional in the copper hydride and gold nanocluster communities, where long-range corrected hybrids are rarely used.

#### The Effect of the Correlation Functional

Throughout this work, we have used the PBE functional with varying amounts of % HF exchange. It would be informative to check the sensitivity of the HOMO–LUMO gap to the nature of the pure functional instead. In Figure 12, we compare the HOMO–LUMO gap for Cu_14_ and Au_20_ using a range of rung 1 local density approximation (LDA), rung 2 generalized gradient approximation (GGA) and rung 3 meta-GGA^131^ functionals. Those functionals are PBE­(GGA), SVWN5­(LDA), SOGGA11­(GGA), XAlpha­(LDA), TPSSTPSS­(meta-GGA), OLYP (GGA), BP86­(GGA), and MN15L­(meta-GGA). All those functionals include 0% HF exchange.

**Figure 12:**
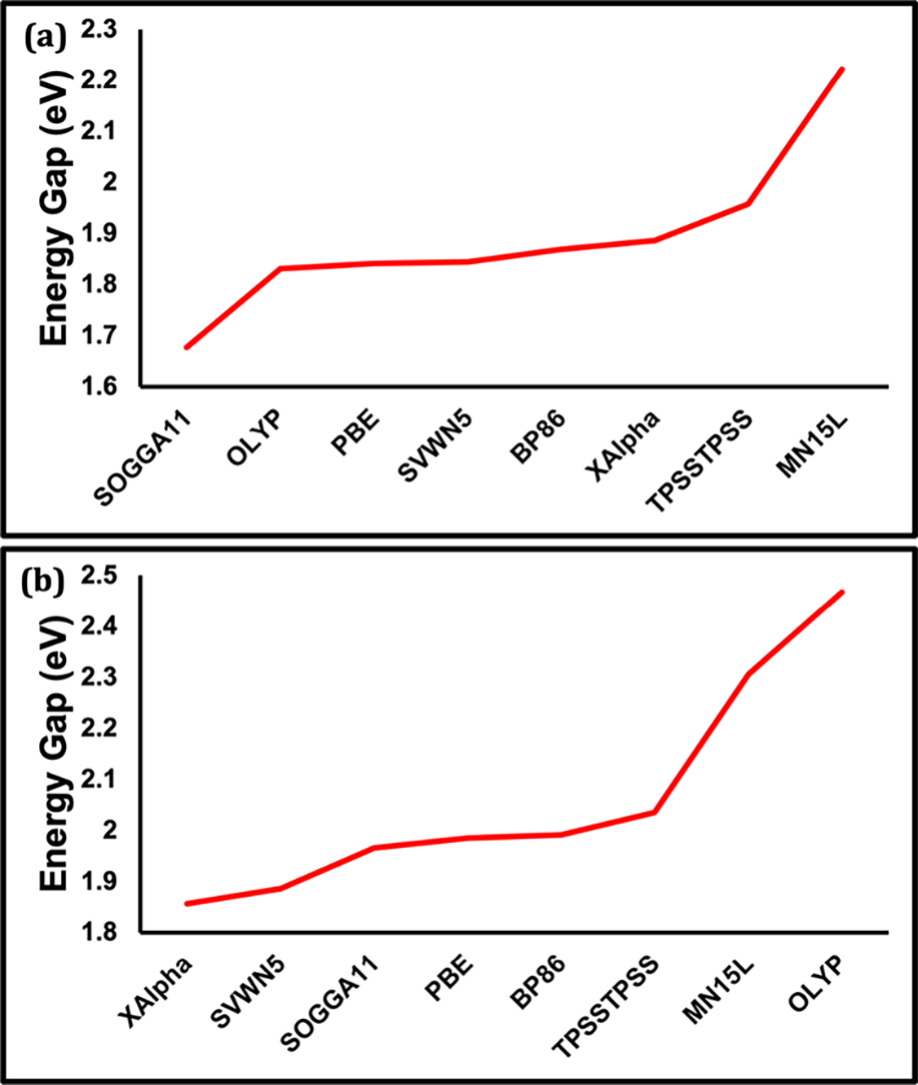
(a) Cu_14_ HOMO–LUMO energy gap computed using eight different pure functionals (XAlpha, SVWN5, SOGGA11, PBE, BP86 TPSSTPSS, MN15L and OLYP). (b) Au_20_ HOMO–LUMO energy gap computed using same set of the eight pure functionals. The functionals are ordered by increasing HOMO–LUMO gap and are therefore not in the same order in the two plots. See Tables S11 and S12 for the energies plotted in panels (a) and (b), respectively.

The HOMO–LUMO gap varies over a range of 0.55 eV in Cu_14_ (from SOGGA11:1.67 eV to MN15L: 2.22 eV). Similarly, the HOMO–LUMO gap varies over a range of 0.61 eV for Au_20_ (from XAlpha: 1.85 eV and OLYP: 2.46 eV) as shown in Figure 12a,b. The magnitude of this range is largely due to a few outliers: MN15L and SOGGA11 for Cu_14_ and MN15L and OLYP for Au_20_. Most other pure functionals are in good agreement, within a range of 0.1 or 0.2 eV of each other. While these errors are not negligible, they are much smaller than the energy changes observed when varying the % HF exchange (a range of 3.9 eV in Cu_14_ and 3.0 eV in Au_20_).

A similar result was recently observed for oscillator strengths of organic compounds where the % HF exchange resulted in a systematic increase in oscillator strength while pure functionals all gave similar strengths.^132^ In general, for both oscillator strengths and energy gaps, the nature of the correlation is not as important as getting the degree of HF exchange right.

#### The Effect of the Basis Set

We study the effect of changing the basis set by increasing order of def2-SV­(P), def2-SVP, def2-SVPD, def2-TZVP and def2-QZVP on the HOMO–LUMO gap in the truncated Cu_14_ and Au_20_ models. The results are shown in Figure 13a for Cu_14_ and Figure 13b for Au_20_. Overall, the effect of the basis set is no larger than 0.06 eV for Cu_14_ and 0.13 eV for Au_20_. This is a considerably smaller effect than the effect of other factors such as the % HF exchange and the nature of the pure functional. While the increase in basis set size results in a decrease in HOMO–LUMO gap in Au_20_, the same systematic change is not observed in Cu_14_. When computing the basis-set dependence of (V)­IE-(V)­EA instead of the HOMO–LUMO gap, we find that the increase of basis set in both Cu_14_ and Au_20_ leads to the systematic decrease in the (V)­IE-(V)­EA energy (see SI Figure S5 and Tables S15 and S16). This means that in both nanoclusters, increasing the basis set size improves the agreement between the computed (V)­IE-(V)­EA energy gap and the experimentally measured electrochemical gap.

**Figure 13:**
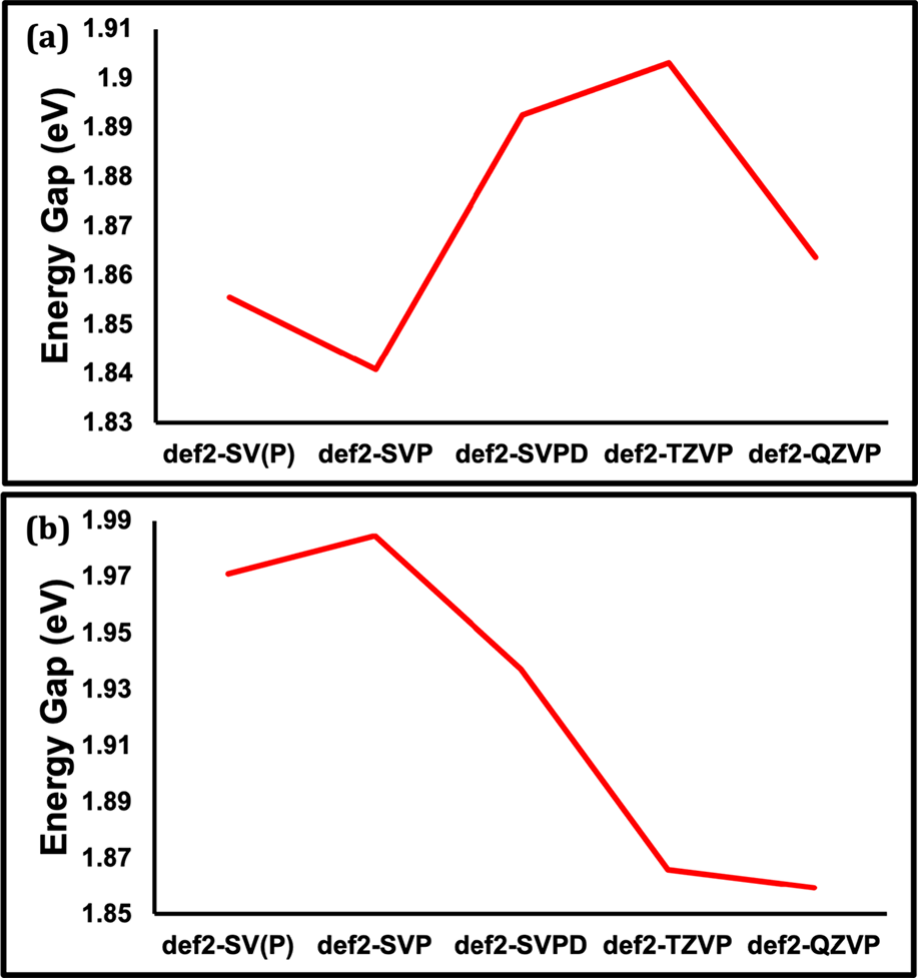
(a) Cu_14_ HOMO–LUMO energy gap computed using five different basis sets (def2-SV­(P), def2-SVP, def2-SVPD, def2-QZVP, def2-TZVP). (b) Au_20_ HOMO–LUMO energy gap values using same set of basis sets. The basis sets are ordered consistently in the two figures by increasing size. See Tables S13 and S14 for the energies plotted in panels (a) and (b), respectively.

## Conclusions and Suggestions for Future DFT Studies on APNCs

We summarize below the key discussions as well as findings in this work and list recommendations for performing computations on APNCs.

(1) The word “band gap” alone, especially when it comes to molecules or small nanoclusters, may refer to any one of several energy gaps that can be computed from first-principles or probed experimentally. Different experimental methods to probe band gaps (UPS-ETS/IPES, UV/vis, or electrochemistry) are not equivalent to each other, and only the fundamental gap is related to the HOMO–LUMO gap in the context of the extended Koopmans’ theorem for exact methods.

(2) The HOMO–LUMO gap should not be used to estimate the magnitude of band gaps unless one is using functionals that are specifically optimized to comply with Koopmans’ theorem such as optimally tuned range-separated functionals. For nontuned functionals, VIE-VEA (e.g., using ΔSCF) is more suitable for computing fundamental gaps, *E*°_ox_ – *E*°_red_ for electrochemical gaps, and VEE (TD-DFT) for optical gaps (if the first excited state is optically active).

(3) (V)­IE-(V)­EA, which employs equilibrium rather than nonequilibrium solvation, may be used as a less computationally demanding approximation to *E*°_ox_ – *E*°_red_ for electrochemical gaps.

(4) While the VIE-VEA, *E*°_ox_ – *E*°_red_, and VEE calculations are not as sensitive to the DFT functional as the HOMO–LUMO gap, we still find substantial errors for the APNCs investigated here when using functionals with varying HF exchange. For example, the magnitude of *E*°_ox_ – *E*°_red_ spans a 1.5 eV range when comparing functionals with 0 and 50% HF exchange. TD-DFT VEEs span a range of 2.4 eV between 0 and 50% HF exchange or with LC-ωPBE. From those calculations, it is immediately clear that some functionals may deviate from experiments by 1 eV or more. Not only are energies affected, but orbital character and solvation energies can also be affected by the functional, resulting in qualitatively incorrect trends.

(5) Regarding the initial choice of functional: For closed-shell/weakly correlated Cu- or Au-based APNCs, the pure PBE functional combined with an appropriate solvation model appears to be a reliable starting point for predicting optical/electrochemical properties, as already established by several researchers in the field. However, how does one select a suitable functional for other (new) systems? Ideally, benchmarking is needed against experimentally determined band gaps for each new class of nanoclusters investigated. This is what led to the wide adoption of the pure PBE functional for gold and copper APNCs,^21,89,90,92,106–116^ in congruence with the results here showing a good agreement with experimental band gaps for 0% or minimal HF exchange.

In the absence of experimental data, benchmarking against more accurate and systematically improvable wave function methods would be the second best option. This may include, for instance, Coupled Cluster (CC)^133^ or Algebraic Diagrammatic Construction (ADC),^134^ which are single-reference methods but can capture an increasing degree of electron correlation through the addition of higher level excitation terms. Excitation energies, ionization energies, and electron affinities can be conveniently obtained by using excitation, annihilation, and creation operators.^135^ However, while modern algorithmic advances have made CC and ADC methods applicable to systems with over a thousand basis functions,^136^ these methods may remain intractable at this time for routine calculations on medium-sized or large nanoclusters.

(6) When the above two options above are not available, it may be tempting to use optimally tuned functionals like range-separated hybrids that have been parametrized to satisfy Koopmans’ theorem. However, if such tuning was carried out in the gas phase for the systems investigated here, it would have led to an optimal functional with a large long-range tuning parameter ω, or with over 50% HF exchange. Such a functional would not have provided quantitative or even qualitative agreement with the experimental data. The reason for this may be the size or complexity of the electronic structure of APNCs; typically, increasing system size results in a smaller “optimal” value of ω for range-separated hybrids.^137–139^

(7) Perform a systematic verification: Before adopting a functional for a new class of APNCs, it is useful to plot key energy metrics (HOMO–LUMO, VEE, (V)­IE-(V)­EA) as a function of the percentage of Hartree–Fock (HF) exchange or ω. This allows one to check the parallelism of these curves and their intersection points relative to experimental values. At the very least, such testing would reveal the sensitivity of these energy gaps to the DFT parameters. The parallelity of different band gaps as a function of exchange or ω is also informative, as nonparallelities can arise between (V)­IE-(V)­EA and VEE from changes in orbital character when modifying the functional. Similarly, the linearity of the HOMO–LUMO gap or other energy gaps as a function of systematically changing the % HF exchange can also be checked. Such testing would also increase confidence in qualitative conclusions derived from the calculations; here, for instance, the observation of MLCT character in Cu_14_ persists up to a high percentage of HF exchange and even for some of the long-range corrected functionals like ωB97XD, which helps to better confirm the MLCT character of the first excited state.

(8) Regarding the solvent model: Nonequilibrium Polarizable Continuum Models (ne-PCM) are appropriate for vertical processes (e.g., absorption spectrum calculations) while Equilibrium PCM (e-PCM) is appropriate for equilibrium processes (e.g., redox potential calculations). Solvation effects are critically important for accurate computation of redox properties; VIE-VEA energies are highly sensitive to the solvent, spanning *a* > 2 eV difference when going from the gas phase to equilibrium solvation (and *a* ∼ 1 eV difference when comparing VIE-VEA energies computed with equilibrium and nonequilibrium solvation models).

(9) Due to the sensitivity of VIE-VEA to the solvent, an energy gap that is less sensitive to solvent, such as VEE, may be a useful alternative as a reference for optimally tuned functionals instead of the fundamental gap, even if Koopmans’ theorem rigorously applies only to the fundamental gap. The fundamental gap and optical gap are closely related such that tuning the HOMO–LUMO gap to the VEE can still give reasonable results. In both systems investigated here, pure functionals give the best agreement between HOMO–LUMO and VEE while also giving the best agreement with experiments, potentially providing a rationale for why pure functionals have been successfully applied to study gold and copper hydride APNCs. However, whether this conclusion continues to apply to other classes of nanoclusters should be further investigated.

(10) The effect of other approximations, such as the nature of the correlation functional, basis set, geometry relaxation, and other contributions to free energy, appear to be substantially smaller than the effect of HF exchange and solvation. Therefore, for studying new classes of APNCs, selecting a functional with a suitable amount of HF exchange or range-separated tuning and using a correct solvation model should generally be the priority before considering other refinements to the computational methods.

## Supplementary Material



## Data Availability

The data supporting this article have been included as part of the Supporting Information.

## Notes

Experimental details including chemicals, synthesis, electrochemical characterization, and spectroscopic measurements of [Cu_14_H_10_(MBN)_3_(TPP)_8_]­BF_4_ and Au_20_(TBBT)_16_; computational details on the approach to tune HF exchange in PBE functional and ω in LC-ωPBE and LRC-ωPBEh; steps for running equilibrium (V)­IE-(V)­EA, nonequilibrium VIE-VEA, (V)­EE, and nonequilibrium VEE; plots of HOMO–LUMO and VEE (TD-DFT/ne-PCM) for truncated and full models of Cu_14_ in solvent and gas phase; tabulated energy gap values for truncated Cu_14_ and Au_20_ in gas phase and solvent for various models (HOMO–LUMO, (V)­EE/TD-DFT, VEE/TD-DFT, (V)­IE-(V)­EA, VIE-VEA, and *E*_ox_ – *E*_red_) as a function of HF exchange; plots of VEE in the gas-phase, ne-PCM, and e-PCM for Cu_14_; tabulated HOMO–LUMO and VEE energy gaps as a function of ω in LC-ωPBE and LRC-ωPBEh for Cu_14_ and Au_20_; tabulated HOMO–LUMO energy gaps for different pure functionals for Cu_14_ and Au_20_; tabulated HOMO–LUMO energy gaps and tabulated and plotted (V)­IE-(V)­EA energy gaps for different basis sets for Cu_14_ and Au_20_; and optimized Cartesian coordinates for [Cu_14_H_10_(MBN)_3_(PH_3_)_8_]^+^, [Cu_14_H_10_(MBN)_3_(PPH_3_)_8_]^+^, and Au_20_(SCH_3_)_16_ (PDF)
