# Supplementary material for: When Do Band Gap Calculations Agree with Experiments in Monolayer-Protected Cu14 and Au20 Atomically Precise Nanoclusters? A (TD)-DFT Comparison of HOMO–LUMO, Fundamental, Optical, and Electrochemical Energy Gaps
Source: J Phys Chem C Nanomater Interfaces. 2026 Apr 13;130(16):5863–81. doi: 10.1021/acs.jpcc.6c01227 (PMC13112356; doi:10.1021/acs.jpcc.6c01227)
Supplement: Supplementary file 1 [file jp6c01227_si_001.pdf]

Supporting Information

When Do Band Gap Calculations Agree with Experiments in Monolayer-Protected Cu_{14} and Au_{20} Atomically Precise Nanoclusters? A (TD)-DFT Comparison of HOMO-LUMO, Fundamental, Optical, and Electrochemical Energy Gaps

Sarah Elhajj[†], Anik Sarkar[†], Yitong Wang[‡], Rongchao Jin[‡], Guoxiang Hu*[¶], Gangli Wang*[†], Samer Gozem*[†]

[†]Department of Chemistry, Georgia State University, Atlanta, GA 30302, USA
[‡]Department of Chemistry, Carnegie Mellon University, Pittsburgh, Pennsylvania 15213, USA.
[¶]School of Materials Science and Engineering, Georgia Institute of Technology, Atlanta, Georgia 30332, United States

Emails: emma.hu@mse.gatech.edu, glwang@gsu.edu, sgozem@gsu.edu

Contents

S1 Experimental Details **S4**

S1.1 Chemicals S4

S1.2 Synthesis S4

S1.2.1 Synthesis of $[Cu(MeCN)_4]BF_4$ S4

S1.2.2 Synthesis of $[Cu_{14}(MBN)_3(TPP)_8H_{10}]BF_4$ Nanoclusters S4

S1.2.3 Synthesis of $Au_{20}(TBBT)_{16}$ S4

S1.3 Electrochemical Characterization S5

S1.3.1 $[Cu_{14}H_{10}(MBN)_3(PPH_3)_8]^+$ Nanoclusters S5

S1.3.2 $Au_{20}(TBBT)_{16}$ Nanoclusters S5

S1.4 Spectroscopic measurement of $[Cu_{14}H_{10}(MBN)_3(TPP)_8]BF_4$ and $Au_{20}(TBBT)_{16}$ S5

S2 Computational Details **S6**

S2.1 Tuning HF exchange in the PBE functional S6

S2.2 Tuning ω in the range separated hybrid LC-ωPBE S6

S2.3 Tuning ω while using 20% short-range PBE exchange S7

S2.4 Running Equilibrium (V)IE-(V)EA and Non-Equilibrium VIE-VEA calculations S7

S2.5 Running Equilibrium (V)EE and Non-Equilibrium VEE calculations S7

S3 Comparison of truncated $[Cu_{14}H_{10}(MBN)_3(PH_3)_8]^+$ vs full $[Cu_{14}H_{10}(MBN)_3(PPH_3)_8]^+$ model system as a function of HF exchange **S8**

S3.1 PCM solvation S8

S3.2 Gas Phase S8

S3.3 Energy Gap results at various HF exchange for a full Cu_{14} model S9

S3.3.1 PCM solvation S9

S3.3.2 Gas Phase S9

S4 Band Gap results at various HF exchange for Cu_{14} truncated model in PCM solvation and Gas Phase **S10**

S5 Band Gap results at various HF exchange fo Au_{20} truncated model in DCM solvent and Gas Phase **S12**

S6 Energy Gap Results for tuning the range-separated parameter ω in LC-ωPBE and LRC-ωPBEh **S13**

S6.0.1 $[Cu_{14}H_{10}(MBN)_3(PH_3)_8]^+$ Truncated model S13

S6.0.2 $Au_{20}(SCH_3)_{16}$ S13

S7 Energy Gap results from different pure functional tests in DCM **S14**

S7.0.1 $[Cu_{14}H_{10}(MBN)_3(PH_3)_8]^+$ S14

S7.0.2 $Au_{20}(SCH_3)_{16}$ S14

S8 (V)IE-(V)EA energy gaps from different basis sets **S15**

S8.0.1 $[Cu_{14}H_{10}(MBN)_3(PH_3)_8]^+$ S15

S8.0.2 $Au_{20}(SCH_3)_{16}$ S16

S8.0.3 $[Cu_{14}H_{10}(MBN)_3(PH_3)_8]^+$ S16

S8.0.4 $Au_{20}(SCH_3)_{16}$ S16

S9 Optimized coordinates at the PBE/def2-SVP level of theory **S16**
S9.1 $[Cu_{14}H_{10}(MBN)_3(PH_3)_8]^+$ S16
S9.2 $[Cu_{14}H_{10}(MBN)_3(PPH_3)_8]^+$ S18
S9.3 $Au_{20}(SCH_3)_{16}$ S25

S1 Experimental Details

S1.1 Chemicals

Cuprous oxide (Cu_2O, ≥99.99%), tetrafluoroboric acid solution (HBF_4, 48 wt%), 4-Mercaptobenzonitrile (MBN, C_7H_5NS, ≥98%), triphenylphosphine (TPP, $C_{18}H_{15}P$, ≥99%), ssodium borohydride ($NaBH_4$, granular, 99.99% metal basis) and Tert-butylammonium perchlorate (TBAP, >99%) were obtained from Sigma-Aldrich. 4-Mercaptobenzonitrile (C_7H_5NS, 95%) was obtained from AmBeed. PLC-grade dichloromethane (≥99.8%, contains amylene as stabilizer) was purchased from Honeywell. HPLC-grade acetonitrile, methanol, and hexane were obtained from Fisher Scientific. Anhydrous dichloromethane (CH_2Cl_2, 99.8%) was purchased from Thermo Scientific. All chemicals were used without further purification. Tert-butyl ammonium perchloride (TBAP, ≥99%), potassium bicarbonate ($KHCO_3$, ≥99%) were purchased from Sigma-Aldrich. Anhydrous dichloromethane (CH_2Cl_2, 99.8%) was purchased from Thermo Scientific, and HPLC-grade dichloromethane (≥99.8%, contains amylene as stabilizer) was purchased from Honeywell. All chemicals were used without any further purification.

S1.2 Synthesis

S1.2.1 Synthesis of $[Cu(MeCN)_4]BF_4$

The synthesis of tetrakis(acetonitrile)copper(I) tetrafluoroborate was carried out following the procedure reported by O. M. Bakr and co-workers.[1] Briefly, 1.0 g of cuprous oxide (Cu_2O) was placed in a round-bottom flask, and 25 mL of acetonitrile was added. The mixture was stirred at 70 °C for 10 min. Subsequently, 5 mL of HBF_4 solution was added dropwise. Upon addition, the red turbid suspension turned into a clear solution. The clear filtrate was cooled to 0 °C and left to crystallize overnight. The resulting crystals were filtered, washed with diethyl ether, and stored under vacuum until further use.

S1.2.2 Synthesis of $[Cu_{14}(MBN)_3(TPP)_8H_{10}]BF_4$ Nanoclusters

$[Cu_{1410}(MBN)_3(TPP)_8H]BF_4$ nanoclusters were synthesized by dissolving 160 mg of $[Cu(MeCN)_4]BF_4$, 20 mg of 4-mercaptobenzonitrile, and 80 mg of triphenylphosphine in 8 mL of acetonitrile under continuous stirring at room temperature. Subsequently, 60 mg of sodium borohydride ($NaBH_4$) in 4 mL of methanol was added dropwise. The reaction mixture was stirred for 1 h, and the precipitate was collected by centrifugation. The solid was redissolved in a 1:1 mixture of dichloromethane and hexane (8 mL) and left undisturbed at 0 °C for crystallization. After 2–3 days, yellow rhombus-shaped crystals were obtained. The crystals were characterized by ESI-MS, SC-XRD, and NMR spectroscopy to confirm cluster composition.

S1.2.3 Synthesis of $Au_{20}(TBBT)_{16}$

The starting nanocluster $[Au_{25}(SC_2H_4Ph)_{18}]^-[TOA]^+$ was synthesized following a previously published procedure [3]. These nanoclusters were then reacted with 0.3 ml of 4-tert-butylbenzenthiol (TBBT) in 1 ml of toluene at 40 oC for 8 hours. Subsequently, approximately 10 ml of methanol was added to quench the reaction, followed by centrifugation at 3000 rpm for 15 minutes. The precipitate was redissolved in 1 ml of DCM and pipetted onto a preparative thin-layer chromatography (PTLC) plate. The mixture was developed with a mixture of DCM and n-hexane (1:3 (v/v)) in a developing tank. The band corresponding to the title nanocluster was cut and dissolved in different solvents for further measurements.

S1.3 Electrochemical Characterization

S1.3.1 $[Cu_{14}H_{10}(MBN)_3(PPH_3)_8]^+$ Nanoclusters

Electrochemical measurements, including cyclic voltammetry (CV) and square-wave voltammetry (SWV), were performed using a CHI 750C potentiostat equipped with a picoamp booster inside a Faraday cage. The analyte solution consisted of 1 mM $[Cu_{14}(MBN)_3(TPP)_8H_{10}]BF_4$ in anhydrous dichloromethane containing 0.1 M TBAP as the supporting electrolyte. A homemade platinum disc electrode (0.6 mm diameter) was used as the working electrode, an Ag/AgCl wire served as a quasi-reference electrode, and a platinum foil was used as the counter electrode. The solution was purged with argon gas for 20–30 min before measurements, and all experiments were conducted under inert atmosphere.

S1.3.2 $Au_{20}(TBBT)_{16}$ Nanoclusters

Electrochemical measurements of $Au_{20}(TBBT)_{16}$ were conducted under the same instrumentation setup as described above. Approximately 0.5 mg of $Au_{20}(TBBT)_{16}$ was dissolved in 0.5 mL of anhydrous dichloromethane containing 0.1 M TBAP as the supporting electrolyte. The same electrode configuration was used (0.6 mm Pt disc working electrode, Pt foil counter electrode, Ag/AgCl quasi-reference electrode). The solution was purged with argon for 20–30 min prior to CV and DPV measurements, and all measurements were carried out under an inert atmosphere.

S1.4 Spectroscopic measurement of $[Cu_{14}H_{10}(MBN)_3(TPP)_8]BF_4$ and $Au_{20}(TBBT)_{16}$

All spectroscopic measurements were conducted using a Shimadzu UV-Vis 1300 spectrophotometer. The measurements were performed in the liquid phase using a 3.5 mL quartz cuvette. The conversion of the absorption spectrum from wavelength (λ) to energy (E) scale was performed using the relation:

$$E\ (\text{eV}) = \frac{1240}{\lambda\ (\text{nm})},$$

which was used as the X-axis. The corresponding absorbance as a function of energy, Abs(E), was calculated using the following transformation:

$$\text{Abs}(E) = \frac{\text{Abs}(\lambda)}{\partial E/\partial \lambda} \propto \text{Abs}(\lambda) \times \lambda^2,$$

and plotted as the Y-axis.[4] To estimate the optical bandgap, a tangent was drawn from the last resolvable absorption band, and the intercept of this tangent with the baseline was considered as the optical bandgap.

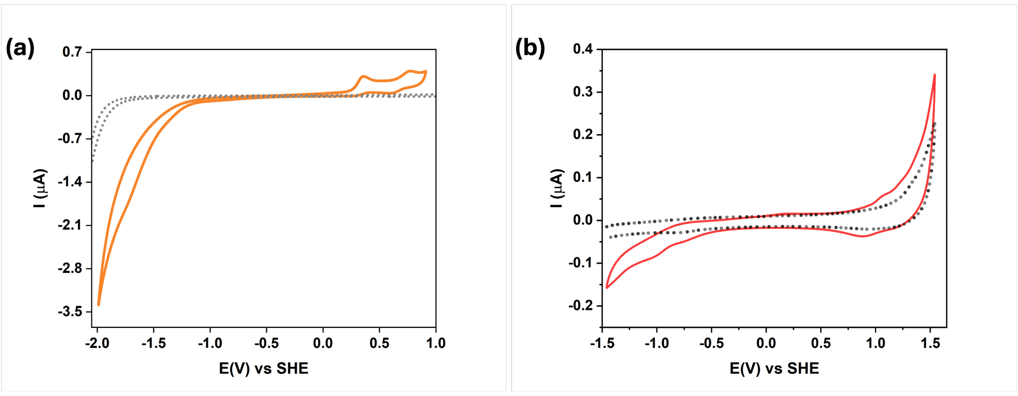

Figure S1: The Cyclic Voltammogram of (a) Cu_{14} and (b) Au_{20} nanoclusters recorded at the scan rate of 0.1 V/s. The grey dashed line in the CV represents the background, collected under identical conditions but without the nanoclusters, for comparison. All potentials are referenced against Standard Hydrogen Electrode potential (SHE).

Table S1: Peak potentials obtained from Differential Pulsed Voltammogram of Cu_{14} and Au_{20} nanoclusters.

**Cu_{14} Nanoclusters**				
**Ox (V)**	0.30_9	0.68_2		
**Red (V)**	0.35_6 (HOMO)	0.69_4	-1.71_7 (LUMO)	
**Au_{20} Nanoclusters**				
**Ox (V)**	1.00_6 (HOMO)	1.18_2	1.34_6	1.50_0
**Red (V)**	-0.82_0 (LUMO)	-1.01_7	-1.19_0	-1.39_0

S2 Computational Details

S2.1 Tuning HF exchange in the PBE functional

To tune %HF exchange in the DFT functional, we utilized Gaussian's IOp keywords with IOP(3/76=1000000xx00) that imposes 10000/10000 (100%) DFT exchange. The last 5 digits, 0xx00/10000 allows for tuning amount of HF exchange with the replacement of xx digits with the desired digits. xx could be hence replaced with 05,10,15,20,25,30,35,40,45,50. Additonally, IOp(3/77=0xx000xx00) was used to tune the DFT exchange from 95% to 50% in increments of 5. To get 35% HF exchange, for instance, we use IOp (3/76=1000003500) and IOp (3/77=0650006500).

S2.2 Tuning ω in the range separated hybrid LC-ωPBE

To tune ω value in the range separated hybrid functional LC-wPBE, we utilized Gaussian's IOp keywords with IOp(3/107=0xxx000000) IOp(3/108=0xxx000000). xxx can be replaced with 001, 010, 100, 200, 300, 400, and 500 for ω values of 0.001, 0.01, 0.1, 0.2, 0.3, 0.4, and 0.5 respectively. For example to obtain an ω value of 0.1, we use the IOp(3/107=0100000000) IOp(3/108=0100000000) which corresponds to 01000/10000. Here ω was tuned in the range, $0.001 < \omega < 0.5\, a_0^{-1}$ where a_0^{-1} denotes the Bohr radius.

S2.3 Tuning ω while using 20% short-range PBE exchange

To tune ω while a functional using what mimics the short-range and long-range behavior of LRC-ωPBEh[2], we utilized Gaussian's IOp keywords with IOp(3/107=0xxx000000) IOp(3/108=0xxx00000[illegible] IOp(3/109=0) IOp(3/119=0800000000) IOp(3/120=0800000000) IOp(3/121=0) IOp(3/130=02000) IOp(3/131=02000). xxx can be replaced with 001, 010, 100, 200, 300, 400, and 500 for ω values of 0.001, 0.01, 0.1, 0.2, 0.3, 0.4, and 0.5 respectively. For example, to obtain an ω value of 0.01, we use the IOp(3/107=0010000000) IOp(3/108=0010000000) which corresponds to 00100/10000. Here ω was tuned in the range, $0.001 < \omega < 0.5\, a_0^{-1}$ where a_0^{-1} denotes the Bohr radius. Note that the IOPs: IOp(3/109=0) IOp(3/119=0800000000) IOp(3/120=0800000000) IOp(3/121=0) IOp(3/130=02000) IOp(3/131=02000) were kept static and only IOp(3/108) and IOp(3/109) were used to tune ω value. To verify that the set of IOPs are applied correctly, we check the HFx and DFx printed lines in Gaussian's log file: HFx wShort= 0.000000 wLong= 0.010000 cFull= 0.200000 cShort= 0.000000 cLong= 0.800000, DFx wShort= 0.000000 wLong= 0.010000 cFull= 0.200000 cShort= 0.000000 cLong= 0.800000

It is important to note that IOp(3/130=02000) sets the full range of HF exchange (HFx cFull= 0.200000), IOp(3/131=02000) sets the full range of DFT exchange (DFx cFull= 0.200000, which means 1-0.2=0.8 DFT exchange is used). IOp(3/119=0800000000) sets the long-range HF exchange to 80% (HFx cLong= 0.800000) and IOp(3/120=0800000000) is used to eliminate the DFT exchange completely. In other words, since we start with 80% DFT at short range IOp(3/131=02000), IOp(3/120=0800000000) makes it 0% at long range by further subtracting those 80% DFT.

S2.4 Running Equilibrium (V)IE-(V)EA and Non-Equilibrium VIE-VEA calculations

To run equilibrium (V)IE-(V)EA, we run single point energy calculations on the Cu_{14} cluster (charge: +1, multiplicity: 1) as well as it's one-electron ionized (2,2) and electron attached (0,2) states using full relaxation of the solvent model with scrf=(pcm, solvent=DiChloroMethane).

To run non-equilibrium VIE-VEA, we first run a single point energy calculation on the Cu_{14} cluster (charge: +1, multiplicity:1) and save the slow/inertial charges to the checkpoint file using the keyword scrf=(pcm,solvent=DiChloroMethane,NonEquilibrium=save). We then run single point calculations for the one-electron ionized (2,2) and electron attached (0,2) states while reading the slow/inertial charges from the checkpoint file of the first calculation of the (1,1) Cu_{14} state, using scrf=(pcm,solvent=DiChloroMethane,NonEquilibrium=Read).

S2.5 Running Equilibrium (V)EE and Non-Equilibrium VEE calculations

To run equilibrium TD-DFT (V)EE calculations for Cu_{14} (1,1) state we use the following keyword TD(Singlets,EqSolv,NStates=30,Root=1) scrf=(pcm,solvent=DiChloroMethane).We note that this is a SP TD-DFT calculation where only the solvent is relaxed around the excited state without geometry optimization of the solute in the excited state. The non-equilibrium VEE TD-DFT calculation is the default option in gaussian when using pcm in combination with the single-point TD-DFT calculation: TD(Singlets,NStates=30) scrf= (pcm,solvent=DiChloroMethane)

S3 Comparison of truncated $[Cu_{14}H_{10}(MBN)_3(PH_3)_8]^+$ vs full $[Cu_{14}H_{10}(MBN)_3(PPH_3)_8]^+$ model system as a function of HF exchange

S3.1 PCM solvation

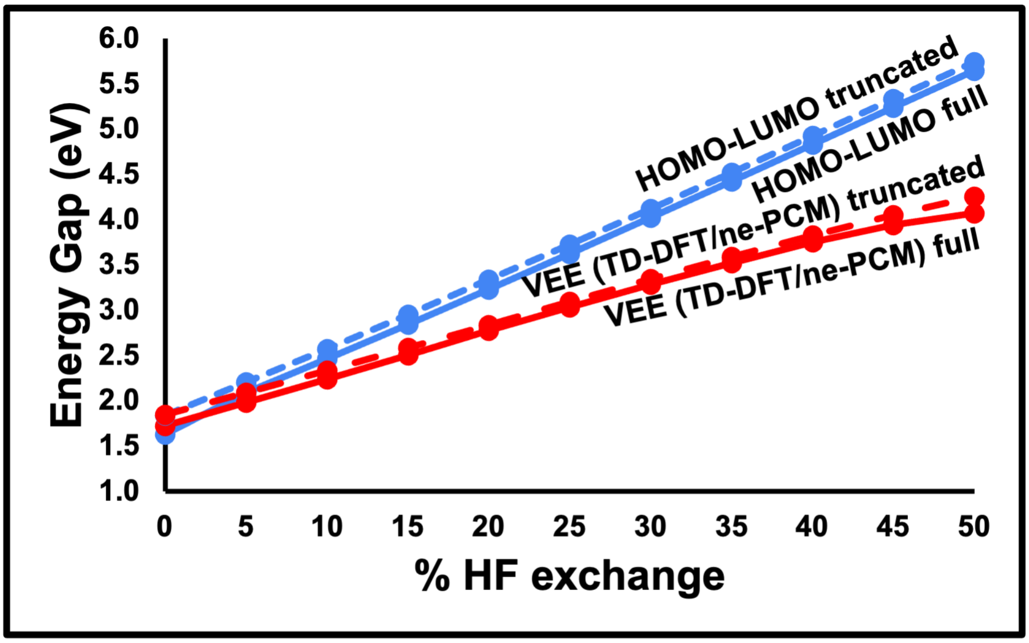

Figure S2: HOMO-LUMO gap (blue) and VEE energies (red) for Cu_{14} computed using two models: the truncated model ($[Cu_{14}H_{10}(MBN)_3(PH_3)_8]^+$, dashed line) and full model ($[Cu_{14}H_{10}(MBN)_3(PPH_3)_8]^+$, solid line).

S3.2 Gas Phase

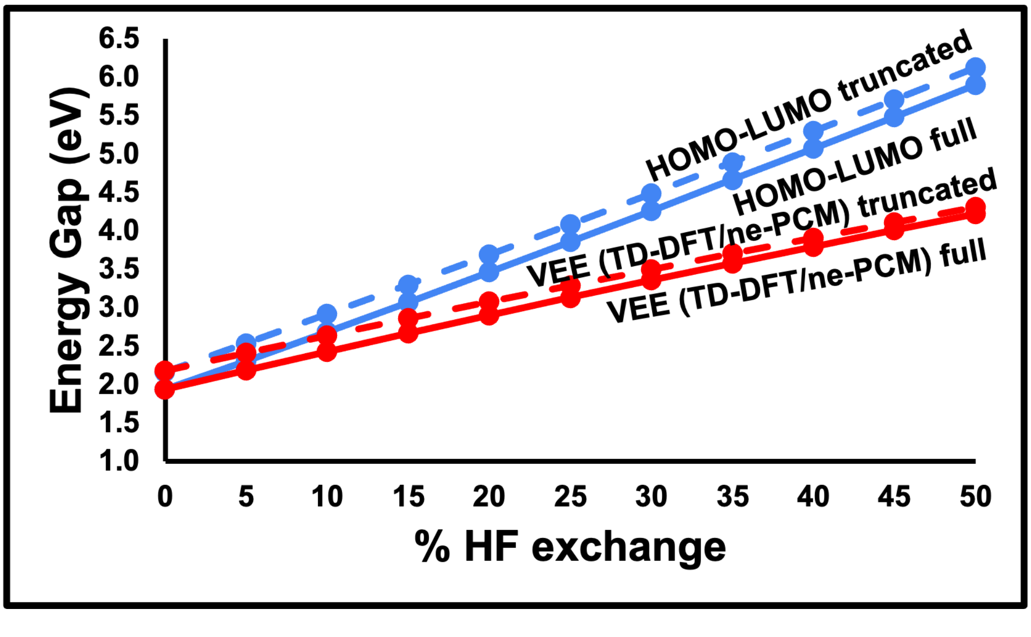

Figure S3: HOMO-LUMO gap (blue) and VEE energies (red) for Cu_{14} computed using two models: the truncated model ($[Cu_{14}H_{10}(MBN)_3(PH_3)_8]^+$, dashed line) and full model ($[Cu_{14}H_{10}(MBN)_3(PPH_3)_8]^+$, solid line).

S3.3 Energy Gap results at various HF exchange for a full Cu_{14} model

S3.3.1 PCM solvation

Table S2: HOMO-LUMO gap and VEE energies predicted at the PBE/def2-SVP level for a full $[Cu_{14}H_{10}(MBN)_3(PPH_3)_8]^+$ model in PCM with DCM solvent at 0%, 5%,10%,15%,20%,25%,30%,35%,40%,45%,50% HF exchange.

HF Exchange (%)	HOMO–LUMO (eV)	VEE (eV)
0	1.62	1.72
5	2.08	1.98
10	2.46	2.24
15	2.84	2.50
20	3.23	2.77
25	3.62	3.03
30	4.02	3.28
35	4.42	3.52
40	4.83	3.75
45	5.23	3.95
50	5.64	4.07

S3.3.2 Gas Phase

Table S3: HOMO-LUMO gap and VEE energies predicted at the PBE/def2-SVP level for a full $[Cu_{14}H_{10}(MBN)_3(PPH_3)_8]^+$ model in gas phase at 0%, 5%,10%,15%,20%,25%,30%,35%,40%,45%,50% HF exchange.

HF Exchange (%)	HOMO–LUMO (eV)	VEE (eV)
0	1.93	1.94
5	2.31	2.19
10	2.68	2.43
15	3.07	2.67
20	3.46	2.90
25	3.86	3.13
30	4.26	3.36
35	4.66	3.58
40	4.83	3.75
45	5.07	3.79
50	5.89	4.22

S4 Band Gap results at various HF exchange for Cu_{14} truncated model in PCM solvation and Gas Phase

Table S4: HOMO-LUMO, VEE, VIE-VEA (ne-PCM) and E_{ox} -E_{red} band gap energies predicted at the PBE/def2-SVP level for a truncated $[Cu_{14}H_{10}(MBN)_3(PH_3)_8]^+$ model in PCM using DCM solvent at 0%, 5%,10%,15%,20%,25%,30%,35%,40%,45%,50% HF exchange.

HF Exchange (%)	HOMO-LUMO (eV)	VEE (eV)	VIE-VEA ne-PCM (eV)	E_{ox} -E_{red} (eV)
0	1.840	1.85	3.33	2.29
5	2.20	2.08	3.51	2.48
10	2.57	2.33	3.68	2.71
15	2.95	2.58	3.86	2.95
20	3.33	2.84	4.04	3.03
25	3.72	3.09	4.22	3.15
30	4.11	3.35	4.42	3.43
35	4.51	3.59	4.59	3.30
40	4.92	3.83	4.76	3.29
45	5.33	4.06	4.87	3.42
50	5.74	4.28	4.98	3.75

Table S5: (V)IE-(V)EA (e-PCM), VIE-VEA (ne-PCM), VIE-VEA (gas) and E_{ox} -E_{red} band gap energies predicted at the PBE/def2-SVP level for a truncated $[Cu_{14}H_{10}(MBN)_3(PH_3)_8]^+$ model in PCM using DCM solvent at 0%, 5%,10%,15%,20%,25%,30%,35%,40%,45%,50% HF exchange.

HF Exchange (%)	(V)IE-(V)EA e-PCM (eV)	VIE-VEA ne-PCM (eV)	VIE-VEA gas (eV)	E_{ox} -E_{red} (eV)
0	2.54	3.33	4.74	2.29
5	2.71	3.51	4.93	2.48
10	2.87	3.68	5.12	2.71
15	3.06	3.86	5.31	2.95
20	3.25	4.04	5.51	3.03
25	3.39	4.22	5.71	3.15
30	3.56	4.42	5.91	3.43
35	3.62	4.59	6.11	3.30
40	3.72	4.76	6.31	3.29
45	3.82	4.87	6.51	3.42
50	3.92	4.98	6.71	3.75

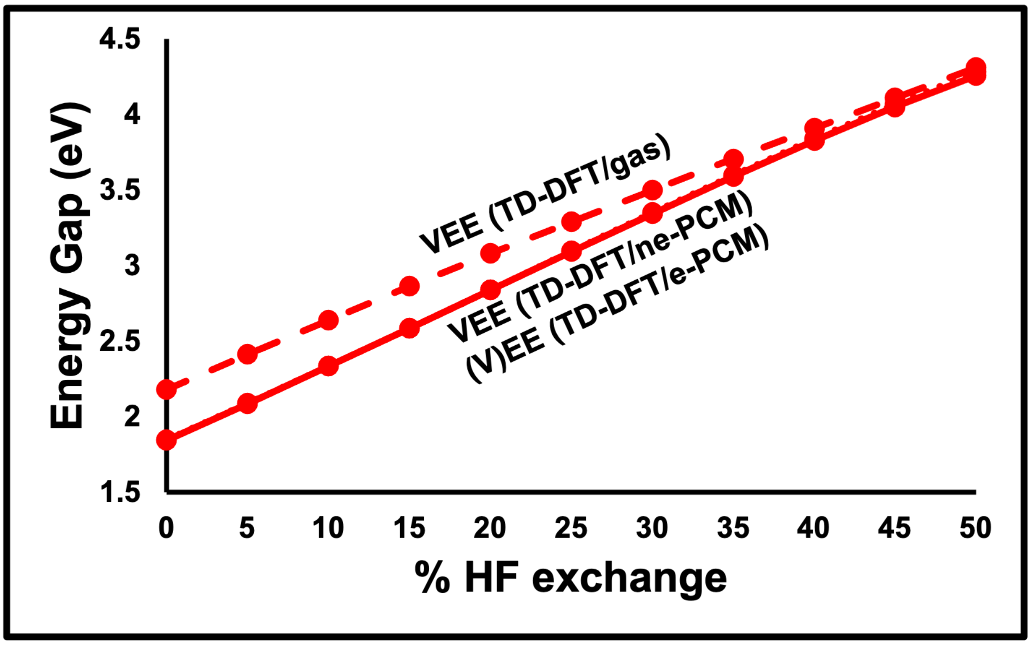

Figure S4: VEE computed band gaps in the gas phase (red dashed line) as a function of varying HF exchange from 0% to 50% for $[Cu_{14}H_{10}(MBN)_3(PH_3)_8]^+$. The same VEE with non-equilibrium solvation (dotted red line) and (V)EE with equilibrium solvation (solid red line).

Table S6: HOMO-LUMO, VEE, and VIE-VEA (ne-PCM) band gap energies predicted at the PBE/def2-SVP level for a truncated $[Cu_{14}H_{10}(MBN)_3(PH_3)_8]^+$ model in gas-phase at 0%, 5%,10%,15%,20%,25%,30%,35%,40%,45%,50% HF exchange.

**HF Exchange (%)**	**HOMO–LUMO (eV)**	**VEE (eV)**	**VIE–VEA (eV)**
0	2.16	2.18	4.74
5	2.53	2.41	4.93
10	2.91	2.64	5.12
15	3.30	2.86	5.31
20	3.69	3.08	5.51
25	4.08	3.29	5.71
30	4.48	3.50	5.91
35	4.89	3.70	6.11
40	5.30	3.91	6.31
45	5.71	4.11	6.51
50	6.12	4.31	6.71

S5 Band Gap results at various HF exchange fo Au_{20} truncated model in DCM solvent and Gas Phase

Table S7: HOMO-LUMO, VEE, (V)IE-(V)EA (e-PCM) and VIE-VEA (ne-PCM) band gap values predicted at the PBE/def2-SVP level for a truncated $Au_{20}(SCH_3)_{16}$ model in PCM using DCM solvent at 0%, 5%,10%,15%,20%,25%,30%,35%,40%,45%,50% HF exchange.

HF Exchange (%)	HOMO-LUMO (eV)	VEE (eV)	(V)IE-(V)EA e-PCM (eV)	VIE-VEA ne-PCM (eV)
0	1.96	2.02	2.38	2.89
5	2.25	2.16	2.51	3.02
10	2.55	2.30	2.64	3.16
15	2.86	2.44	2.77	3.29
20	3.16	2.58	2.90	3.42
25	3.47	2.72	3.02	3.54
30	3.77	2.84	3.12	3.65
35	4.06	2.94	3.22	3.75
40	4.34	3.04	3.31	3.84
45	4.63	3.13	3.39	3.93
50	4.92	3.22	3.48	4.02

Table S8: HOMO-LUMO, VEE, and VIE-VEA band gap values predicted at the PBE/def2-SVP level for a truncated $Au_{20}(SCH_3)_{16}$ model in gas-phase at 0%, 5%,10%,15%,20%,25%,30%,35%,40%,45%,50% HF exchange.

HF Exchange (%)	HOMO–LUMO (eV)	VEE (eV)	VIE–VEA (eV)
0	1.93	1.99	4.30
5	2.22	2.12	4.43
10	2.52	2.27	4.57
15	2.82	2.41	4.71
20	3.13	2.55	4.85
25	3.44	2.69	4.99
30	3.74	2.82	5.10
35	4.03	2.93	5.20
40	4.31	3.03	5.30
45	4.59	3.12	5.39
50	4.88	3.21	5.48

S6 Energy Gap Results for tuning the range-separated parameter ω in LC-ωPBE and LRC-ωPBEh

S6.0.1 $[Cu_{14}H_{10}(MBN)_3(PH_3)_8]^+$ Truncated model

Table S9: Comparison of HOMO–LUMO and VEE values for LC-ωPBE//PBE and LRC-ωPBEh//PBE with def2-SVP basis set at various ω values. LC-ωPBE//PBE and LRC-ωPBEh//PBE entails that the energies are reported at LC-ωPBE, LRC-ωPBEh levels respectively with the PBE optimized structure.

ω (a_0^{-1})	LC–ωPBE$_{\textbf{geom PBE}}$		LRC–ωPBEh$_{\textbf{geom PBE}}$	
	**HOMO–LUMO (eV)**	**VEE (eV)**	**HOMO–LUMO (eV)**	**VEE (eV)**
0.001	1.888	1.862	3.655	3.164
0.01	2.164	1.864	3.876	3.165
0.1	4.522	2.462	5.822	3.722
0.2	6.163	3.167	7.242	4.385
0.3	7.182	3.727	8.129	4.685
0.4	7.849	4.184	8.704	4.845
0.5	8.312	4.555	9.092	4.927

S6.0.2 $Au_{20}(SCH_3)_{16}$

Table S10: Comparison of HOMO–LUMO and VEE values for LC-ωPBE//PBE and LRC-ωPBEh//PBE with def2-SVP basis set at various ω values. LC-ωPBE//PBE and LRC-ωPBEh//PBE entails that the energies are reported at LC-ωPBE, LRC-ωPBEh levels respectively with the PBE optimized structure.

ω (a_0^{-1})	LC–ωPBE$_{\textbf{geom PBE}}$		LRC–ωPBEh$_{\textbf{geom PBE}}$	
	**HOMO–LUMO (eV)**	**VEE (eV)**	**HOMO–LUMO (eV)**	**VEE (eV)**
0.001	1.991	2.021	1.991	2.793
0.01	2.268	2.022	2.268	2.794
0.1	4.506	2.334	4.506	2.947
0.2	5.891	2.898	5.891	3.279
0.3	6.664	3.263	6.664	3.542
0.4	7.128	3.507	7.128	3.712
0.5	7.418	3.670	7.418	3.814

S7 Energy Gap results from different pure functional tests in DCM

S7.0.1 $[Cu_{14}H_{10}(MBN)_3(PH_3)_8]^+$

Table S11: HOMO-LUMO band gap energies computed with different exchange-correlation functionals using a def2-SVP basis set for $[Cu_{14}H_{10}(MBN)_3(PH_3)_8]^+$ in DCM solvent.

DFT Functional	HOMO-LUMO (eV)
SOGGA11	1.67
OLYP	1.83
PBEPBE	1.84
SVWN5	1.84
BP86	1.87
XAlpha	1.88
TPSSTPSS	1.95
MN15L	2.22

S7.0.2 $Au_{20}(SCH_3)_{16}$

Table S12: HOMO-LUMO band gap energies computed with different exchange-correlation functionals using a def2-SVP basis set for $Au_{20}(SCH_3)_{16}$ in DCM solvent.

DFT Functional	HOMO-LUMO (eV)
XAlpha	1.85
SVWN5	1.88
SOGGA11	1.96
PBEPEBE	1.98
BP86	1.99
TPSSTPSS	2.03
MN15L	2.30
OLYP	2.46

S8 (V)IE-(V)EA energy gaps from different basis sets

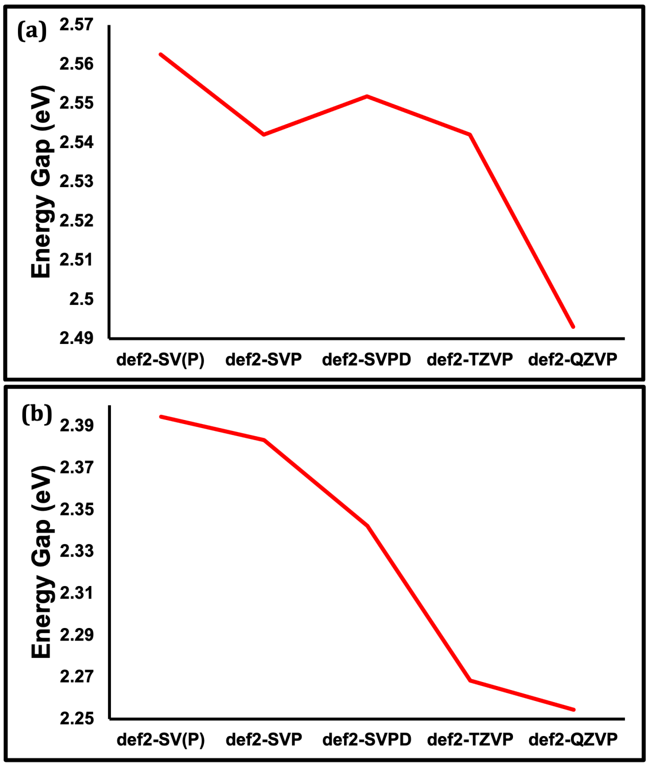

Figure S5: (a) Cu_{14} (V)IE-(V)EA (e-PCM) energy gaps computed using five different basis sets (def2-SV(P), def2-SVP, def2-SVPD, def2-QZVP, def2-TZVP). (b) Au_{20} (V)IE-(V)EA energy gaps computed using same set of basis sets.

S8.0.1 $[Cu_{14}H_{10}(MBN)_3(PH_3)_8]^+$

Table S13: HOMO-LUMO energy gaps computed with different basis sets at the PBE level for $[Cu_{14}H_{10}(MBN)_3(PH_3)_8]^+$ in DCM solvent.

Basis set	HOMO-LUMO (eV)
def2-SV(P)	1.85
def2-SVP	1.84
def2-SVPD	1.89
def2-TZVP	1.90
def2-QZVP	1.86

S8.0.2 $Au_{20}(SCH_3)_{16}$

Table S14: HOMO-LUMO energy gaps computed with different basis sets at the PBE level for $Au_{20}(SCH_3)_{16}$ in DCM solvent

Basis set	HOMO-LUMO (eV)
def2-SV(P)	1.97
def2-SVP	1.98
def2-SVPD	1.94
def2-TZVP	1.87
def2-QZVP	1.86

S8.0.3 $[Cu_{14}H_{10}(MBN)_3(PH_3)_8]^+$

Table S15: (V)IE-(V)EA (e-PCM) energy gaps computed with different basis sets at the PBE level for $[Cu_{14}H_{10}(MBN)_3(PH_3)_8]^+$ in DCM solvent.

Basis set	(V)IE-(V)EA (eV)
def2-SV(P)	2.56
def2-SVP	2.54
def2-SVPD	2.55
def2-TZVP	2.54
def2-QZVP	2.49

S8.0.4 $Au_{20}(SCH_3)_{16}$

Table S16: (V)IE-(V)EA (e-PCM) energy gaps computed with different basis sets at the PBE level for $Au_{20}(SCH_3)_{16}$ in DCM solvent.

Basis set	(V)IE-(V)EA (eV)
def2-SV(P)	2.39
def2-SVP	2.38
def2-SVPD	2.34
def2-TZVP	2.27
def2-QZVP	2.25

S9 Optimized coordinates at the PBE/def2-SVP level of theory

S9.1 $[Cu_{14}H_{10}(MBN)_3(PH_3)_8]^+$

```
Cu                 0.6073400000        1.5510160000        0.5042340000
H                  1.5316130000        1.5633420000        1.9980700000
H                  0.7555500000       -0.0877920000       -0.1362650000
H                  1.7947200000        2.4741800000       -0.6930850000
Cu                 0.4632770000        0.5220270000        2.9481470000
Cu                 2.3458180000        0.0612700000        1.4457160000
```

H	3.8540790000	0.4125120000	0.7636510000
Cu	3.5701170000	-0.6464110000	-0.6758820000
Cu	3.2050900000	1.9615740000	0.2323050000
Cu	-3.1024830000	0.6059610000	0.6576250000
P	-5.2023370000	1.0532030000	1.2640250000
P	5.8259610000	-1.0639870000	-1.1428400000
P	4.6273470000	3.6898200000	0.5869890000
P	0.8384320000	0.9501740000	5.1502310000
S	-1.6244990000	-0.3419630000	2.2864460000
C	-2.2256400000	-1.7103450000	3.2543240000
C	-3.5263750000	-2.2122110000	3.0216990000
C	-4.0177710000	-3.2855030000	3.7681370000
C	-3.2124460000	-3.8848510000	4.7687410000
C	-1.9046300000	-3.3864510000	4.9978060000
H	-1.2721000000	-3.8492530000	5.7692460000
C	-1.4195160000	-2.3146550000	4.2464080000
H	-0.3999150000	-1.9373990000	4.4170950000
C	-3.7115170000	-4.9845830000	5.5368080000
N	-4.1205020000	-5.8884840000	6.1681040000
Cu	-0.1791800000	-0.3120550000	-1.6208680000
H	0.4625010000	0.9281210000	-2.7012990000
H	0.6434090000	-1.8961600000	-2.3507630000
Cu	-0.1896770000	2.3082420000	-1.8417230000
Cu	1.8959990000	1.0342360000	-1.6140910000
H	3.1812600000	0.1424690000	-2.2591300000
Cu	2.0066460000	-0.9610620000	-2.9682310000
P	2.4902160000	-1.5182280000	-5.1107810000
P	-0.3184280000	4.1936780000	-3.1021180000
S	-1.5675650000	2.3302680000	0.0671380000
C	-1.7737990000	3.9947700000	0.6647840000
C	-3.0645070000	4.4909560000	0.9539490000
C	-3.2380030000	5.8001290000	1.4088960000
C	-2.1151210000	6.6472620000	1.5824340000
C	-0.8191660000	6.1515490000	1.2912320000
H	0.0568260000	6.8024410000	1.4269200000
C	-0.6536450000	4.8405510000	0.8399390000
H	0.3542240000	4.4537030000	0.6212380000
C	-2.2867300000	7.9897630000	2.0487330000
N	-2.4265800000	9.0928330000	2.4311170000
Cu	0.1912000000	-1.2265970000	1.0715600000
H	0.3768350000	-2.8303760000	0.3515050000
H	1.6796860000	-1.2466380000	2.3236590000
Cu	-0.9172660000	-2.6554290000	-0.8233640000
Cu	1.4703430000	-2.0816540000	-0.8663830000
H	3.0769500000	-2.3632180000	-0.4124640000
Cu	2.5540370000	-2.3688920000	1.2708020000
P	3.4659410000	-4.0689790000	2.4623520000
P	-1.6823710000	-4.6494590000	-1.5949910000
S	-2.4101660000	-0.8563120000	-1.0869200000
C	-3.3866080000	-1.0288870000	-2.5654070000

C	-4.7540210000	-0.6740490000	-2.5547940000
C	-5.5347360000	-0.8171220000	-3.7040980000
C	-4.9596470000	-1.3236550000	-4.8962990000
C	-3.5871000000	-1.6791640000	-4.9087930000
H	-3.1330180000	-2.0683610000	-5.8316940000
C	-2.8121900000	-1.5305440000	-3.7568420000
H	-1.7439650000	-1.7974930000	-3.7719040000
C	-5.7548710000	-1.4718590000	-6.0773000000
N	-6.4073350000	-1.5942390000	-7.0478290000
H	-4.1452660000	-1.7544050000	2.2353620000
H	-5.0306350000	-3.6698970000	3.5794750000
H	-3.9385150000	3.8381230000	0.8139390000
H	-4.2465150000	6.1777470000	1.6311170000
H	-5.2018710000	-0.2815050000	-1.6297680000
H	-6.5980980000	-0.5377730000	-3.6862200000
H	-5.4273900000	1.7390850000	2.5003840000
H	-6.1488210000	0.0006730000	1.4822710000
H	6.6578790000	-1.7440410000	-0.1946980000
H	6.7299910000	0.0190720000	-1.3940990000
H	5.4636940000	4.1120780000	-0.4949490000
H	5.6488800000	3.6482630000	1.5890470000
H	0.6477460000	2.3021440000	5.5771370000
H	0.1106770000	0.3274270000	6.2146500000
H	1.4300290000	-1.7243610000	-6.0495840000
H	3.1804420000	-2.7478230000	-5.3556350000
H	0.8297130000	4.6767280000	-3.8057070000
H	-0.7221220000	5.4454350000	-2.5366890000
H	3.8788920000	-5.2933640000	1.8459900000
H	2.7399470000	-4.6560930000	3.5467930000
H	-0.7837520000	-5.6024880000	-2.1701070000
H	-2.6989670000	-4.7309150000	-2.5995580000
H	2.1470510000	0.7742770000	5.7012470000
H	6.2076950000	-1.8514950000	-2.2775580000
H	3.3061030000	-0.6922120000	-5.9482880000
H	-1.2204810000	4.2021220000	-4.2126800000
H	-2.2945040000	-5.5321120000	-0.6501460000
H	-6.0524800000	1.8755720000	0.4562500000
H	4.6786760000	-3.8195470000	3.1802420000
H	4.1101910000	4.9820770000	0.9199410000

S9.2 $[Cu_{14}H_{10}(MBN)_3(PPH_3)_8]^+$

Cu	-0.6035020000	-1.0038900000	-1.3559180000
H	0.1461990000	-0.2362000000	-2.7335400000
H	-0.0184310000	0.0009530000	-0.0034440000
H	0.6901160000	-2.5059720000	-1.1401430000
Cu	-0.7825630000	1.2829610000	-2.7673340000
Cu	1.2707520000	0.6355880000	-1.5599220000
H	2.7628420000	-0.1617110000	-1.7301300000

Cu	3.1318310000	0.0215110000	0.0379980000
Cu	1.8467000000	-1.6395490000	-2.1733770000
Cu	-3.9442640000	-0.0389980000	-0.0588360000
P	-6.1951940000	-0.1102320000	-0.1001920000
P	5.4409880000	0.0205020000	0.0899000000
P	2.6533130000	-2.8214420000	-3.9515370000
P	-0.8950200000	2.3137510000	-4.8030380000
S	-2.6785380000	1.5710100000	-1.3159810000
C	-3.4486660000	3.1781050000	-1.4378080000
C	-4.6903490000	3.3081820000	-2.1022620000
C	-5.3108530000	4.5538140000	-2.2156720000
C	-4.6995710000	5.7043160000	-1.6576460000
C	-3.4603820000	5.5747600000	-0.9837560000
H	-2.9778370000	6.4601090000	-0.5456970000
C	-2.8463220000	4.3250210000	-0.8771560000
H	-1.8825280000	4.2275800000	-0.3529860000
C	-5.3287880000	6.9849880000	-1.7695910000
N	-5.8448580000	8.0380050000	-1.8594410000
C	-7.0744720000	1.5189040000	-0.0956950000
C	-8.0345070000	1.8792850000	-1.0630670000
H	-8.2882540000	1.1810620000	-1.8746770000
C	-8.6773420000	3.1276920000	-0.9928610000
H	-9.4246130000	3.3976420000	-1.7549620000
C	-8.3776260000	4.0207030000	0.0475900000
H	-8.8875790000	4.9943480000	0.1057990000
C	-7.4206510000	3.6663250000	1.0150660000
H	-7.1847970000	4.3595250000	1.8370010000
C	-6.7620370000	2.4307980000	0.9370360000
H	-6.0003810000	2.1680550000	1.6887420000
C	-1.3981810000	4.0939520000	-4.8237030000
C	-2.1869510000	4.6575900000	-5.8500040000
C	-2.4869360000	6.0294060000	-5.8370380000
H	-3.1047750000	6.4588270000	-6.6406880000
C	-2.0023200000	6.8497750000	-4.8046110000
H	-2.2398520000	7.9245740000	-4.7964340000
C	-1.2254600000	6.2923090000	-3.7756210000
H	-0.8544410000	6.9237390000	-2.9539630000
C	-0.9321500000	4.9197400000	-3.7789250000
H	-0.3468050000	4.4822000000	-2.9545740000
C	0.6742680000	2.3198330000	-5.7825940000
C	1.7522630000	1.5270830000	-5.3377330000
H	1.6509050000	0.9424020000	-4.4078890000
C	2.9539750000	1.4914440000	-6.0650870000
H	3.7878830000	0.8703720000	-5.7070960000
C	3.0885360000	2.2475820000	-7.2403200000
H	4.0299830000	2.2164400000	-7.8100690000
C	2.0234730000	3.0525940000	-7.6819010000
H	2.1277540000	3.6562720000	-8.5965100000
C	0.8237100000	3.0947010000	-6.9546490000
H	0.0013730000	3.7384760000	-7.3024150000

C	-2.1357450000	1.5205880000	-5.9197580000
C	-1.8204710000	0.9822810000	-7.1840630000
H	-0.7881250000	1.0243450000	-7.5614560000
C	-2.8244600000	0.3940150000	-7.9742170000
H	-2.5651730000	-0.0212590000	-8.9602920000
C	-4.1500860000	0.3459320000	-7.5156710000
H	-4.9360100000	-0.1049070000	-8.1405730000
C	-4.4692540000	0.8771420000	-6.2531830000
H	-5.5066750000	0.8470280000	-5.8868390000
C	-3.4683210000	1.4470450000	-5.4536720000
C	1.3878850000	-3.2654370000	-5.2262360000
C	1.3297160000	-4.5365640000	-5.8359680000
H	2.0399530000	-5.3233520000	-5.5413820000
C	0.3675570000	-4.8053150000	-6.8246310000
H	0.3333640000	-5.8009720000	-7.2930630000
C	-0.5403560000	-3.8086480000	-7.2172930000
H	-1.2900510000	-4.0191280000	-7.9953610000
C	-0.4926370000	-2.5429040000	-6.6075300000
H	-1.2056530000	-1.7575750000	-6.9015840000
C	0.4579110000	-2.2742040000	-5.6105160000
H	0.4726780000	-1.2855280000	-5.1245950000
C	3.9843800000	-2.0025850000	-4.9410800000
C	4.9599580000	-1.2665960000	-4.2349160000
H	4.8758020000	-1.1582130000	-3.1422130000
C	6.0299300000	-0.6690290000	-4.9190870000
H	6.7838520000	-0.0984350000	-4.3554890000
C	6.1280750000	-0.7883480000	-6.3165310000
H	6.9640810000	-0.3149160000	-6.8537950000
C	5.1512240000	-1.5063930000	-7.0262680000
H	5.2189920000	-1.5971270000	-8.1212690000
C	4.0833530000	-2.1136060000	-6.3439260000
H	3.3246370000	-2.6762340000	-6.9086860000
C	3.4164410000	-4.4459320000	-3.5113000000
C	4.4624430000	-5.0245480000	-4.2615430000
H	4.8842460000	-4.4900500000	-5.1260480000
C	4.9704840000	-6.2850840000	-3.9084500000
H	5.7865450000	-6.7281320000	-4.4998850000
C	4.4383890000	-6.9784600000	-2.8082920000
H	4.8356990000	-7.9682350000	-2.5353800000
C	3.4042470000	-6.4023100000	-2.0517330000
H	2.9903860000	-6.9328600000	-1.1819390000
C	2.9020940000	-5.1371090000	-2.3949180000
H	2.1112970000	-4.6729030000	-1.7848770000
C	6.1860490000	0.1490400000	1.7792750000
C	7.4098050000	-0.4621100000	2.1283430000
H	7.9568720000	-1.0650980000	1.3876590000
C	7.9335760000	-0.3086590000	3.4227490000
H	8.8877420000	-0.7913020000	3.6847650000
C	7.2422240000	0.4531510000	4.3790670000
H	7.6511440000	0.5677790000	5.3946180000

C	6.0189800000	1.0562520000	4.0413880000
H	5.4611420000	1.6356690000	4.7925020000
C	5.4884640000	0.9001340000	2.7510880000
H	4.5167620000	1.3530340000	2.4961110000
C	-6.8763840000	-0.9602580000	-1.5942320000
C	-8.0750370000	-1.7040750000	-1.5776230000
H	-8.6428350000	-1.8176360000	-0.6418420000
C	-8.5499850000	-2.3059100000	-2.7546270000
H	-9.4849560000	-2.8865980000	-2.7298980000
C	-7.8364700000	-2.1686060000	-3.9567120000
H	-8.2080110000	-2.6445620000	-4.8771360000
C	-6.6381990000	-1.4354970000	-3.9775930000
H	-6.0621920000	-1.3443070000	-4.9104620000
C	-6.1545010000	-0.8417550000	-2.8016420000
H	-5.1972150000	-0.2954340000	-2.8112340000
C	-6.9930780000	-0.9816320000	1.3217610000
C	-8.2935500000	-0.6658990000	1.7719830000
H	-8.8678790000	0.1347510000	1.2814820000
C	-8.8576000000	-1.3638770000	2.8519620000
H	-9.8723670000	-1.1098730000	3.1949330000
C	-8.1279580000	-2.3768190000	3.4957600000
H	-8.5663110000	-2.9168420000	4.3488800000
C	-6.8301220000	-2.6883460000	3.0577960000
H	-6.2456450000	-3.4616040000	3.5779440000
C	-6.2621360000	-1.9945470000	1.9775480000
H	-5.2344390000	-2.2201800000	1.6499120000
C	6.2491200000	1.4010940000	-0.8407990000
C	7.4749830000	1.9816260000	-0.4496910000
H	7.9887170000	1.6292680000	0.4577010000
C	8.0435380000	3.0153380000	-1.2123310000
H	8.9985430000	3.4627770000	-0.8964310000
C	7.3956540000	3.4786870000	-2.3693140000
H	7.8398480000	4.2925100000	-2.9626970000
C	6.1707640000	2.9116450000	-2.7593740000
H	5.6467840000	3.2851900000	-3.6521360000
C	5.5955050000	1.8834070000	-1.9962120000
H	4.6222140000	1.4574070000	-2.2888090000
C	6.2396680000	-1.4955340000	-0.6090470000
C	7.4693600000	-1.4701030000	-1.3014650000
H	7.9903830000	-0.5148580000	-1.4669820000
C	8.0334400000	-2.6619850000	-1.7862250000
H	8.9915700000	-2.6298520000	-2.3275280000
C	7.3779120000	-3.8875230000	-1.5840050000
H	7.8190410000	-4.8199930000	-1.9681660000
C	6.1490760000	-3.9189060000	-0.9032530000
H	5.6190850000	-4.8727650000	-0.7608110000
C	5.5774360000	-2.7296770000	-0.4247650000
H	4.6010960000	-2.7542200000	0.0856560000
Cu	-0.6458440000	-0.6828860000	1.5234470000
H	0.1193360000	-2.2486270000	1.5865300000

H	0.6010560000	0.2689490000	2.7507860000
Cu	-0.7684340000	-3.0513600000	0.2726870000
Cu	1.2694810000	-1.6677510000	0.2561320000
H	2.7344110000	-1.4125570000	1.0784120000
Cu	1.7577810000	-1.0670760000	2.5527410000
P	2.4738630000	-1.9930600000	4.5092280000
P	-0.8449150000	-5.3291250000	0.4365990000
S	-2.6443210000	-1.9649330000	-0.7585250000
C	-3.3450270000	-2.8981840000	-2.1115930000
C	-4.4329750000	-3.7688180000	-1.8730080000
C	-4.9920010000	-4.5111110000	-2.9147890000
C	-4.4787310000	-4.3862690000	-4.2296100000
C	-3.3988460000	-3.5029810000	-4.4741980000
H	-2.9933440000	-3.4003480000	-5.4908320000
C	-2.8397380000	-2.7714120000	-3.4239760000
H	-1.9932780000	-2.0918750000	-3.6115770000
C	-5.0467440000	-5.1446750000	-5.3022330000
N	-5.5125610000	-5.7666450000	-6.1851430000
C	-1.4035500000	-6.2415820000	-1.0718530000
C	-2.2070840000	-7.4004450000	-1.0150390000
C	-2.5688990000	-8.0670930000	-2.1973070000
H	-3.1977270000	-8.9690930000	-2.1428610000
C	-2.1328350000	-7.5852180000	-3.4426000000
H	-2.4199790000	-8.1079360000	-4.3678290000
C	-1.3420220000	-6.4254820000	-3.5055380000
H	-1.0086500000	-6.0316240000	-4.4778140000
C	-0.9859420000	-5.7507100000	-2.3277300000
H	-0.3905800000	-4.8253230000	-2.3856290000
C	0.7431520000	-6.1685680000	0.8786150000
C	1.7856260000	-5.3934360000	1.4282250000
H	1.6597600000	-4.3026240000	1.5317560000
C	2.9845270000	-6.0036080000	1.8336140000
H	3.7890510000	-5.3885320000	2.2626420000
C	3.1535050000	-7.3902890000	1.6912270000
H	4.0921610000	-7.8665610000	2.0135860000
C	2.1271290000	-8.1665830000	1.1250830000
H	2.2592890000	-9.2519990000	0.9978520000
C	0.9297260000	-7.5594810000	0.7135910000
H	0.1380340000	-8.1747960000	0.2593480000
C	-2.0322090000	-5.9017310000	1.7335770000
C	-1.7374300000	-6.8938820000	2.6914160000
H	-0.7403140000	-7.3569780000	2.7208520000
C	-2.7163160000	-7.2993280000	3.6158510000
H	-2.4725430000	-8.0732180000	4.3597960000
C	-3.9984830000	-6.7284830000	3.5862380000
H	-4.7670010000	-7.0563960000	4.3026690000
C	-4.2949380000	-5.7323460000	2.6393850000
H	-5.2976950000	-5.2806270000	2.6076840000
C	-3.3149100000	-5.3090720000	1.7298350000
C	1.1500010000	-2.8558610000	5.4708910000

C	0.9967190000	-2.7096120000	6.8657340000
H	1.6625090000	-2.0368570000	7.4265950000
C	-0.0032210000	-3.4245980000	7.5472200000
H	-0.1129830000	-3.3019610000	8.6357620000
C	-0.8509580000	-4.2975280000	6.8463770000
H	-1.6283360000	-4.8624920000	7.3833660000
C	-0.7080770000	-4.4420150000	5.4554320000
H	-1.3706280000	-5.1201400000	4.8959080000
C	0.2771190000	-3.7164500000	4.7684410000
H	0.3653420000	-3.8181860000	3.6751860000
C	3.8109370000	-3.2647250000	4.3871920000
C	4.8439730000	-3.0332260000	3.4537010000
H	4.8003540000	-2.1476370000	2.8004280000
C	5.9205870000	-3.9279460000	3.3514150000
H	6.7200930000	-3.7334280000	2.6203000000
C	5.9678550000	-5.0706540000	4.1691620000
H	6.8092210000	-5.7755240000	4.0853590000
C	4.9332930000	-5.3151570000	5.0876280000
H	4.9612180000	-6.2122850000	5.7250080000
C	3.8587730000	-4.4165990000	5.2001080000
H	3.0552610000	-4.6146790000	5.9255330000
C	3.1715010000	-0.7810770000	5.7173230000
C	4.1753710000	-1.1253940000	6.6475070000
H	4.6017640000	-2.1398700000	6.6508890000
C	4.6363090000	-0.1751490000	7.5730180000
H	5.4198640000	-0.4535360000	8.2945340000
C	4.0985340000	1.1228780000	7.5800700000
H	4.4587810000	1.8656810000	8.3083730000
C	3.1050540000	1.4736740000	6.6504620000
H	2.6869820000	2.4907610000	6.6423730000
C	2.6497370000	0.5293810000	5.7167000000
H	1.8902490000	0.8129280000	4.9707790000
Cu	-0.6310440000	1.6687350000	-0.1994270000
H	0.0962890000	2.4943420000	1.1539510000
H	0.6554780000	2.2550780000	-1.5886140000
Cu	-0.8397660000	1.7467380000	2.4707370000
Cu	1.2328440000	1.0513500000	1.3427420000
H	2.7221440000	1.6290780000	0.7600900000
Cu	1.7941430000	2.7344190000	-0.3093570000
P	2.5710840000	4.8763450000	-0.4368490000
P	-1.0600040000	3.0728090000	4.3167620000
S	-2.7046970000	0.3058210000	1.9937630000
C	-3.4439490000	-0.3769490000	3.4699840000
C	-4.4226820000	0.3652060000	4.1696960000
C	-5.0345850000	-0.1606590000	5.3088770000
C	-4.6769040000	-1.4501910000	5.7743780000
C	-3.6951350000	-2.1950710000	5.0761110000
H	-3.4085920000	-3.1939090000	5.4345870000
C	-3.0861560000	-1.6601850000	3.9383650000
H	-2.3203790000	-2.2370750000	3.3965740000

C	-5.2948860000	-1.9939140000	6.9451830000
N	-5.8000650000	-2.4431920000	7.9076310000
C	-1.7535560000	2.2805190000	5.8352530000
C	-2.6732540000	2.9178630000	6.6940120000
C	-3.1340310000	2.2605600000	7.8475260000
H	-3.8529970000	2.7657180000	8.5108430000
C	-2.6819390000	0.9665350000	8.1529180000
H	-3.0470590000	0.4529560000	9.0555310000
C	-1.7735590000	0.3228460000	7.2950570000
H	-1.4250160000	-0.6974760000	7.5174190000
C	-1.3181650000	0.9714250000	6.1377750000
H	-0.6270200000	0.4503690000	5.4558030000
C	0.4823790000	3.8880020000	4.9328650000
C	1.5766690000	3.9962430000	4.0482420000
H	1.5131420000	3.5425510000	3.0451910000
C	2.7470030000	4.6634380000	4.4455670000
H	3.5927020000	4.7412920000	3.7464450000
C	2.8372510000	5.2229550000	5.7308110000
H	3.7540070000	5.7481280000	6.0399290000
C	1.7617890000	5.0960140000	6.6269810000
H	1.8340460000	5.5170720000	7.6415390000
C	0.5916770000	4.4250870000	6.2347700000
H	-0.2375400000	4.3154270000	6.9507510000
C	-2.2034550000	4.4859730000	3.9672090000
C	-2.1102480000	5.7448900000	4.5985240000
H	-1.3138950000	5.9408570000	5.3317750000
C	-3.0266890000	6.7633790000	4.2868160000
H	-2.9439940000	7.7406450000	4.7866570000
C	-4.0375430000	6.5394970000	3.3371660000
H	-4.7501610000	7.3409780000	3.0892970000
C	-4.1257010000	5.2937130000	2.6934460000
H	-4.8980800000	5.1190750000	1.9298420000
C	-3.2138780000	4.2723400000	3.0035280000
C	1.2824800000	6.1804660000	-0.1872840000
C	1.2492350000	7.3819290000	-0.9262330000
H	1.9860580000	7.5611100000	-1.7235540000
C	0.2772540000	8.3579400000	-0.6463680000
H	0.2614360000	9.2920160000	-1.2287650000
C	-0.6641000000	8.1469580000	0.3745200000
H	-1.4197450000	8.9160140000	0.5967740000
C	-0.6426840000	6.9476160000	1.1076930000
H	-1.3812320000	6.7705550000	1.9042080000
C	0.3178690000	5.9649390000	0.8215440000
H	0.3143380000	5.0182340000	1.3844200000
C	3.8813230000	5.3500530000	0.7814800000
C	4.8497550000	4.3768310000	1.1082220000
H	4.7768090000	3.3682890000	0.6721190000
C	5.8976510000	4.6866550000	1.9891470000
H	6.6451810000	3.9171590000	2.2354110000
C	5.9818750000	5.9674950000	2.5624260000

H	6.8006000000	6.2086730000	3.2575890000
C	5.0123450000	6.9358430000	2.2526820000
H	5.0681290000	7.9381110000	2.7045680000
C	3.9654420000	6.6314530000	1.3662220000
H	3.2105480000	7.3971790000	1.1320950000
C	3.3354100000	5.3116280000	-2.0624290000
C	4.3485020000	6.2851180000	-2.1957540000
H	4.7457650000	6.7960270000	-1.3055920000
C	4.8576380000	6.6043090000	-3.4650540000
H	5.6478410000	7.3650970000	-3.5589280000
C	4.3606210000	5.9578130000	-4.6094860000
H	4.7588850000	6.2126290000	-5.6036510000
C	3.3614760000	4.9783230000	-4.4815100000
H	2.9767700000	4.4569540000	-5.3705960000
C	2.8585850000	4.6489110000	-3.2128050000
H	2.0980860000	3.8590060000	-3.1084640000
H	-5.1804620000	2.4185660000	-2.5237170000
H	-6.2775620000	4.6422880000	-2.7314120000
H	-2.5758870000	4.0235890000	-6.6613230000
H	-3.7221040000	1.8470640000	-4.4594810000
H	-4.8489790000	-3.8580170000	-0.8590140000
H	-5.8346500000	-5.1894930000	-2.7187290000
H	-2.5592710000	-7.7823290000	-0.0445840000
H	-3.5402000000	-4.5093230000	1.0064680000
H	-4.6975110000	1.3705730000	3.8182580000
H	-5.7910570000	0.4256300000	5.8498670000
H	-3.0382710000	3.9300810000	6.4623700000
H	-3.2711400000	3.3017400000	2.4830970000

S9.3 $Au_{20}(SCH_3)_{16}$

Au	3.6822960000	3.5592120000	-0.0868910000
Au	0.9840600000	2.4857250000	0.9975920000
Au	-2.0972360000	3.3189650000	1.4152980000
Au	1.9493600000	-1.6189520000	1.4648290000
Au	-4.1769110000	-2.6606550000	1.4228570000
Au	2.4042180000	0.8913800000	3.4432720000
Au	0.5093610000	5.2829020000	-0.1611960000
Au	-0.7216810000	-2.3994940000	1.2120750000
S	3.4016870000	-1.2477710000	3.3848200000
S	4.8740350000	1.9378950000	1.1672710000
S	2.5685800000	5.2441970000	-1.3280000000
S	1.3488960000	2.9969360000	3.3818550000
S	-2.6543100000	1.1345960000	2.1178070000
S	-2.3460720000	-3.2614110000	2.7813600000
S	-6.0960550000	-2.2949920000	0.1025440000
S	-1.5786860000	5.5755270000	0.9226630000
Au	3.5793800000	-3.7000090000	0.0128770000
Au	0.9278970000	-2.5129080000	-1.0049690000
Au	-2.1776870000	-3.2661460000	-1.4135800000

Au	1.9538510000	1.5276710000	-1.4742960000
Au	-4.1719150000	2.7175920000	-1.4786050000
Au	2.3091920000	-0.9476640000	-3.4815100000
Au	0.3635570000	-5.3314650000	0.1202840000
Au	-0.6965690000	2.3690310000	-1.1982100000
S	3.3581430000	1.1669950000	-3.4328900000
S	4.8018600000	-2.1128210000	-1.2556620000
S	2.4431920000	-5.3740910000	1.2503330000
S	1.1775780000	-3.0090300000	-3.4007070000
S	-2.5702050000	-1.0433910000	-2.1199040000
S	-2.3005060000	3.1020660000	-2.8548390000
S	-6.1538970000	2.4046500000	-0.2365770000
S	-1.6954040000	-5.5499460000	-1.0239930000
Au	4.6305250000	-0.0843840000	-0.0437450000
Au	0.1694950000	-0.0141400000	0.0301130000
Au	-2.8443310000	0.0592730000	-0.0135240000
Au	-6.0629180000	0.0544710000	-0.0646440000
C	-2.8889550000	-6.1592380000	0.2384710000
H	-2.6117570000	-7.2006200000	0.4888100000
H	-2.8729620000	-5.5323380000	1.1481580000
H	-3.8984990000	-6.1393340000	-0.2133140000
C	3.2135930000	-6.9651510000	0.7241330000
H	4.2478410000	-6.9868710000	1.1160980000
H	2.6282850000	-7.7899340000	1.1724140000
H	3.2248680000	-7.0661290000	-0.3754000000
C	-2.3139530000	-2.0660350000	4.1821260000
H	-2.3117600000	-1.0229820000	3.8143890000
H	-3.1965050000	-2.2500700000	4.8232910000
H	-1.3895540000	-2.2589060000	4.7592120000
C	2.6086690000	-2.2205290000	4.7364910000
H	3.0364010000	-1.8934050000	5.7030330000
H	1.5129520000	-2.0808480000	4.7427390000
H	2.8453750000	-3.2880370000	4.5664010000
C	6.5631770000	-2.5921140000	-0.9794970000
H	7.2016150000	-1.8356290000	-1.4733030000
H	6.8060230000	-2.6456180000	0.0965720000
H	6.7227770000	-3.5791130000	-1.4529230000
C	6.6457530000	2.3648740000	0.8724220000
H	6.8405360000	3.3450570000	1.3468310000
H	7.2666850000	1.5878890000	1.3566180000
H	6.8779240000	2.4145560000	-0.2061900000
C	3.3823180000	6.8293110000	-0.8529350000
H	3.4241910000	6.9517960000	0.2436670000
H	4.4067050000	6.8211390000	-1.2704360000
H	2.8031690000	7.6572070000	-1.3034550000
C	-1.1281400000	6.2756990000	2.5673220000
H	-2.0575880000	6.3652940000	3.1604950000
H	-0.4095930000	5.6218900000	3.0947490000
H	-0.6896810000	7.2785280000	2.4074510000
C	-7.5152820000	2.7445190000	-1.4336550000

H	-7.5030560000	3.8273970000	-1.6587270000
H	-7.3891580000	2.1649220000	-2.3648210000
H	-8.4712890000	2.4764600000	-0.9461230000
C	-7.5179810000	-2.6826600000	1.2104600000
H	-7.4598140000	-2.1171000000	2.1569080000
H	-8.4488360000	-2.4245900000	0.6715150000
H	-7.4968690000	-3.7688760000	1.4181890000
C	-4.3249290000	1.2634560000	2.8746720000
H	-4.2067840000	1.7602180000	3.8566180000
H	-5.0191880000	1.8405040000	2.2382190000
H	-4.7134360000	0.2359140000	3.0149710000
C	-2.1587620000	4.9364420000	-2.9619270000
H	-1.1792950000	5.1714680000	-3.4197210000
H	-2.9732000000	5.3080930000	-3.6122590000
H	-2.2244870000	5.4083540000	-1.9652730000
C	2.5360190000	2.1691580000	-4.7456560000
H	1.4384210000	2.0491400000	-4.7136090000
H	2.7989570000	3.2300900000	-4.5724770000
H	2.9231670000	1.8451590000	-5.7302020000
C	2.4441310000	-4.3304440000	-3.5892560000
H	3.3350930000	-4.1209990000	-2.9686200000
H	1.9817310000	-5.2834150000	-3.2670540000
H	2.7286460000	-4.3962190000	-4.6564880000
C	-4.1871120000	-1.0632890000	-2.9941560000
H	-4.4944700000	-0.0114990000	-3.1543750000
H	-4.0295500000	-1.5636700000	-3.9686190000
H	-4.9614590000	-1.5953320000	-2.4130470000
C	2.6868820000	4.2534580000	3.5197290000
H	2.2662670000	5.2299820000	3.2106210000
H	3.0126900000	4.3067470000	4.5758190000
H	3.5431170000	3.9967940000	2.8687090000

References

[1] Atanu Ghosh et al. "[Cu61 (StBu) 26S6Cl6H14]+: A Core–Shell Superatom Nanocluster with a Quasi-J 36 Cu19 Core and an "18-Crown-6" Metal-Sulfide-like Stabilizing Belt". In: *ACS Materials Letters* 1.3 (2019), pp. 297–302.

[2] Mary A Rohrdanz, Katie M Martins, and John M Herbert. "A long-range-corrected density functional that performs well for both ground-state properties and time-dependent density functional theory excitation energies, including charge-transfer excited states". In: *The Journal of chemical physics* 130.5 (2009).

[3] Zhikun Wu et al. "Kinetic control and thermodynamic selection in the synthesis of atomically precise gold nanoclusters". In: *Journal of the American Chemical Society* 133.25 (2011), pp. 9670–9673.

[4] Manzhou Zhu, Huifeng Qian, and Rongchao Jin. "Thiolate-protected Au20 clusters with a large energy gap of 2.1 eV". In: *Journal of the American Chemical Society* 131.21 (2009), pp. 7220–7221.
